# Previous prescribed burns saved thousands of ancient sequoias during historically unprecedented wildfires

**DOI:** 10.1038/s41467-026-75418-6

**Published:** 2026-07-11

**Authors:** Dan J. Dixon, Adrian J. Das, Xiaoli Dong, Andrew M. Latimer, David N. Soderberg, Nathan L. Stephenson, Anthony C. Caprio, Yufang Jin

**Affiliations:** 1 Department of Land, Air and Water Resources, University of California Davis, Davis, CA USA; 2 U.S. Geological Survey, Western Ecological Research Center, Sequoia and Kings Canyon Field Station, Three Rivers, CA USA; 3 Department of Environmental Science and Policy, University of California Davis, Davis, CA USA; 4 Department of Plant Sciences, University of California Davis, Davis, CA USA; 5 Division of Resources Management and Science, Sequoia and Kings Canyon National Parks, Three Rivers, CA USA

**Keywords:** Fire ecology, Natural hazards, Forest ecology

## Abstract

Wildfires in California’s Sierra Nevada during 2020–2021 killed giant sequoias (*Sequoiadendron giganteum*) at rates unseen for millennia, underscoring the vulnerability of highly fire-adapted trees to ongoing environmental change. Following a century of fire exclusion and fuel accumulation, the effectiveness of prescribed burns in reducing giant sequoia mortality from wildfire remained poorly quantified. Here we estimate mortality outcomes for 26,403 giant sequoias across 19 groves in Sequoia and Kings Canyon national parks following the Castle (2020) and KNP Complex (2021) wildfires using a Bayesian framework. We map tree mortality using a deep learning classifier integrating 3 m PlanetScope imagery, airborne lidar, and field observations. From an estimated 7,974 sequoia deaths (95% Bayesian credible interval (CI): 7,555–8,430), corresponding to 30.2% mortality (CI: 28.6–31.9%), we find previous prescribed burns (≤10 years prior) reduced mortality odds by 77% (CI: 69–83%), making treated trees nearly four times more likely to survive. Counterfactual simulations suggest that prescribed burns prevented at least 1,888 (CI: 1,487–2,302) deaths, and universal treatment would have saved an additional 3,888 (CI: 3,236–4,580) giant sequoias. These results show that prescribed burns substantially improve survival during extreme wildfires, offering guidance for conserving long-lived, fire-adapted forests under intensifying fire regimes.

## Abstract (web summary)

Wildfires during 2020–2021 killed giant sequoias at unprecedented rates in California’s Sierra Nevada. Dixon et al. estimate prescribed burns saved nearly 2,000 giant sequoias, and that broader treatment could have saved thousands more.

## Introduction

Wildfires are increasing in size and severity across the western United States (U.S.), threatening even the most fire-adapted mature and old-growth forests^1–5^. These shifts are driven by rising temperatures, intensifying drought, extreme fire weather, and more than a century of fire exclusion^6–10^. As a result, forests once considered resistant now experience widespread, high severity fire, causing extensive mortality of iconic overstory trees and threatening associated biodiversity^1,3,4,11,12^. This contrasts sharply with historical regimes, such as those in California’s Sierra Nevada, where frequent low- to moderate-severity fires reduced fuels, opened canopies, and maintained heterogeneous forest structures that promoted resilience to future fire^10,11,13,14^. These shifts are part of a global trend in temperate, frequent-fire forests, where altered fire regimes are exceeding the adaptive capacity of long-lived trees^5^.

Among the most striking examples of this emerging vulnerability is the giant sequoia (*Sequoiadendron giganteum* L. Buchholz)^1,11^. Though sequoias can survive for over 3000 years, recent fires killed an estimated 13–19% of mature individuals in 2020–2021, losses unmatched over millennia of climatic and fire variability^1,11,15^. Their adaptations including thick bark, elevated crowns, and fire-facilitated seed release evolved under a regime of frequent low to moderate severity fire^11,16^. Yet recent extreme wildfires have overwhelmed these adaptations, killing seed-bearing mature trees^1^, destroying cones^17^, and reducing regeneration of some patches to well below historical levels^11,17^. Understanding how management can affect mortality risk in remaining populations is now critical.

While prescribed burning is widely used to reduce wildfire risk in fire-adapted forests^10,14^, empirical evidence of its effectiveness in reducing giant sequoia mortality has been limited. Sequoia and Kings Canyon national parks (SEKI) implement one of the most extensive prescribed fire programs in the U.S., treating sequoia populations (“groves”) since the 1960s^18^. Prior to the 2020–2021 wildfires, this program produced a mosaic of fire histories within and between groves: some areas were treated with prescribed burns while others had not burned for over a century^19^. Despite decades of implementation, treatment effectiveness remained anecdotal, largely because mortality events of this magnitude had not been observed previously^11^. This gap creates uncertainty for managers who must balance ecological benefits against operational constraints, including risk, resources, and regulations, without clear evidence of treatment efficacy^20^. More broadly, quantifying prescribed fire effects on species-level outcomes remains challenging across heterogeneous fire weather, topographic, and forest conditions^21,22^.

Pre-fire forest structure can also affect fire behavior and subsequent mortality^23^. Following fire exclusion, many Sierra Nevada mixed-conifer forests exhibit denser canopies, fewer gaps, and greater fuel continuity, which increase the likelihood of crown fire^14,24,25^. In similar forest types, managers often apply mechanical thinning treatments to reduce these structural characteristics and restore forest resilience^10,26^. While thinning treatments were limited in SEKI groves, natural variation in pre-fire structure provides an opportunity to test whether structure specifically influences giant sequoia mortality. Key structural metrics, including canopy density, height heterogeneity, and ladder fuels (vertically continuous live or dead vegetation facilitating upward fire movement into tree crowns), can be quantified at landscape scales using airborne lidar collected across SEKI groves in 2015–2016, providing high-resolution covariates for evaluating structural effects on mortality^27^.

Although multiple large-scale studies have assessed prescribed burning and forest structure effects on post-fire severity, these assessments rely primarily on coarse-resolution satellite-derived metrics that are not designed to resolve species-specific mortality outcomes at the scale of individual trees^26,28–30^. In the Sierra Nevada, commonly used fire severity metrics derived from 30 m Landsat imagery quantify aggregated post-fire spectral change, with treated forests often exhibiting lower severity values and reduced basal area loss^26,28,29,31,32^. However, these metrics aggregate outcomes across mixed pixels that contain multiple species and vegetation types, rather than resolving mortality of individual trees^26^. As a result, severity classes correspond to wide and overlapping mortality ranges, limiting inference about treatment effectiveness for long-lived, fire-resistant species such as giant sequoia^1,33^. Tree-level mortality datasets at finer spatial resolution are therefore needed to evaluate species-specific outcomes under extreme wildfire conditions.

Recent advances in fine-scale satellite imagery and deep learning enable tree-level post-fire mortality mapping across large landscapes^34,35^. PlanetScope time series (3 m resolution) collected before and after fire can be classified with a spatio-temporal 3D convolutional neural network (CNN) that learns spectral changes between pre- and post-fire imagery to distinguish live from dead tree crowns^34^. Trained on over one million fire-affected trees in California, this approach generalizes across diverse fire, forest, and illumination conditions and, when integrated with pre-fire lidar and mapped stems, can resolve individual tree outcomes needed for species-specific inference.

**Fig. 1:**
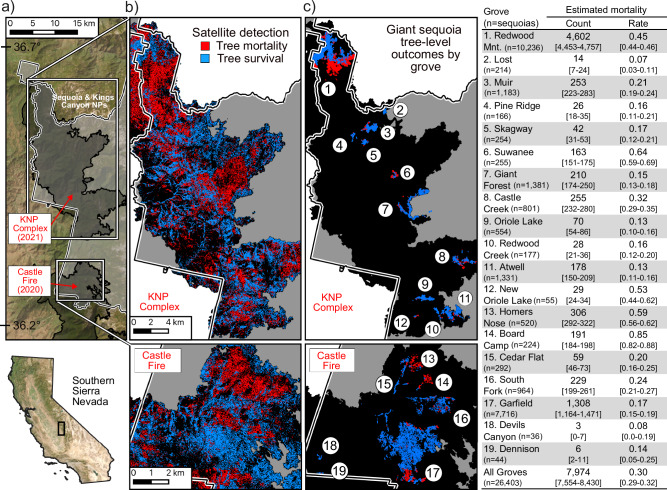
Study area, mortality mapping, and grove-level posterior mortality estimates. **a** Study area in the southern Sierra Nevada, California, within Sequoia and Kings Canyon national parks (black with white stroke) and the KNP and Castle fire perimeters (black lines); **b** PlanetScope-derived tree mortality and survival classification (majority class) in the first year post-fire from Dixon et al.^34^; and **c** locations of the 19 giant sequoia groves included in this study, colored by calibrated tree-level posterior mortality probability, where trees with a posterior mortality probability greater than 0.5 (*p* > 0.5, red) were classified as dead and trees with a posterior mortality probability less than 0.5 (*p* < 0.5, blue) were classified as alive. Here, *p* denotes the calibrated posterior probability of post-fire tree mortality derived from ground observations. We follow the Sequoia Tree Inventory categorization of Garfield Grove, which excludes Dillonwood Grove. Includes copyrighted material of Planet Labs PBC. All rights reserved.

Here we provide species-specific, landscape-scale estimates of giant sequoia mortality by integrating CNN-based tree mortality estimation with management history and pre-fire forest structure. We assemble 26,403 mapped giant sequoia stems, lidar-derived neighborhood structure, and PlanetScope imagery to classify trees as live or dead based on >90% loss of photosynthetic crown foliage as observed in aerial imagery 1 year post-fire (Fig. 1). We ask: (1) How many giant sequoias died in Sequoia and Kings Canyon (SEKI) national parks following the 2020–2021 Castle and KNP Complex fires? (2) How did prescribed burn history and local forest structure affect mortality? (3) How many additional sequoias would have survived or died under counterfactual scenarios of more or less prescribed fire? Our approach quantifies the landscape-scale influence of prescribed burning and structure on sequoia survival, demonstrating how forest management can enhance resilience under intensifying wildfire regimes.

## Results

### Workflow overview

Our analysis included a calibration phase followed by a three-step Bayesian workflow (Supplementary Fig. 1). In the calibration phase, we adjusted CNN-derived mortality probabilities against an independent dataset to ensure sequoia-specific accuracy. Step 1 of the Bayesian workflow estimated tree-level mortality probabilities for all mapped stems (>20 m tall) using the calibrated 3D-CNN applied to 3 m PlanetScope imagery^34^. Step 2 modeled how prescribed burning and lidar-derived forest structure influenced mortality, among other covariates. Prescribed fire treatment was represented at the tree level as a binary variable distinguishing trees that experienced a prescribed burn prior to wildfire. Step 3 simulated counterfactual prescribed burn histories by modifying the prescribed burn treatment indicator to estimate survival or mortality under alternative management scenarios. Uncertainty was propagated with Monte Carlo sampling across all modeling steps, including calibration and model estimation.

### Post-fire tree mortality calibration and assessment

Because the CNN was trained across multiple tree species, we calibrated its outputs with a Bayesian logistic model using an independent dataset of 1566 sequoias (813 dead); the spatial distribution of these field observations is shown in Supplementary Fig. 2. This produced species-specific posterior mortality probabilities and ensured that satellite-derived uncertainty was propagated through downstream analyses (Supplementary Methods). Across five-fold cross-validation on the field dataset, the calibration phase yielded a mean F1 score of 0.844, compared to 0.813 for the uncalibrated model (Supplementary Methods).

Applying the calibrated model to all 26,403 mapped sequoias, we generated 6000 Bernoulli mortality outcomes per tree from posterior distributions. Across the 19 groves, we estimated a median of 7974 dead sequoias (95% CI, Bayesian credible interval: 7555–8430), corresponding to 30.2% mortality (95% CI: 28.6–31.9%). Redwood Mountain grove (KNP Complex) experienced the largest absolute loss: 4602 deaths (95% CI: 4453–4757) or 45% mortality (95% CI: 44–46%), while Board Camp Grove exhibited the most severe proportional loss, with 85% mortality (95% CI: 82.1–88.4%), corresponding to 191 deaths (95% CI: 184–198). These findings highlight both the scale of the wildfire-caused mortality and the heterogeneity of impacts across groves. Posterior predictive checks and additional calibration details are provided in Supplementary Methods.

### Drivers of mortality

**Fig. 2:**
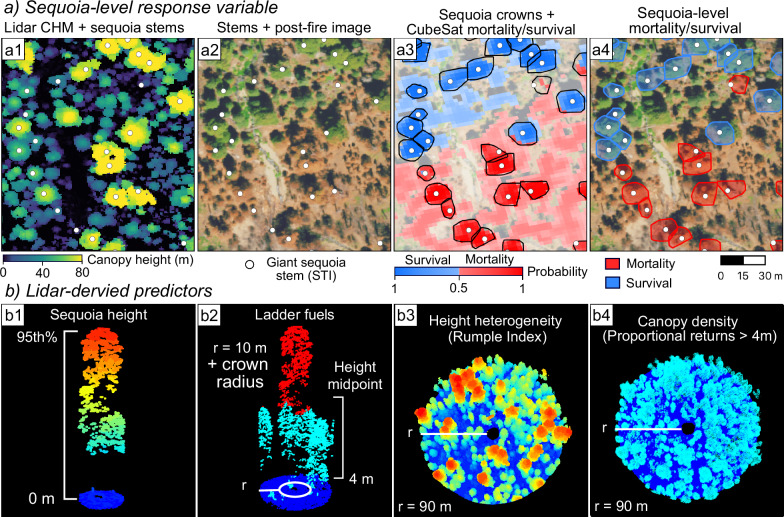
Tree-level mortality mapping and lidar-derived structural predictors. **a** Examples of tree-level giant sequoia mortality/survival response variable and **b** lidar-derived predictors. Sequoia stem locations (STI data, white points) at Redwood Mountain grove are overlaid with **a1** pre-fire aerial lidar-derived canopy height model (CHM) at 1 m resolution; **a2** post-fire aerial image in 2022 from the National Agriculture Imagery Program (0.6 m); **a3** CubeSat (PlanetScope)-derived tree mortality and survival probability maps (3 m) with tree-level giant sequoia crown polygons (segmented from airborne lidar); **a4** sequoia crowns with example mortality/survival simulated outcome. Subplot **b** shows **b1** the sequoia height at the 95th percentile; **b2** ladder fuels within 10 m of the crown footprint; and **b3**–**4** show the heterogeneity of canopy heights and density of lidar returns over 4 m.

We assessed how prescribed fire and forest structure influenced sequoia mortality using an ensemble of logistic regression models fit to spatially balanced random subsets of sequoias (3501 trees) to reduce fine-scale spatial autocorrelation. Each model resampled trees to capture selection variability while retaining Step 1 mortality uncertainty. Fixed effects included a binary indicator of recent prescribed burning (≤10 years pre-fire) and lidar-derived metrics of canopy density, height heterogeneity, and ladder fuels (Fig. 2). Additional covariates included topography (heat load index, slope, topographic position), a binary indicator of upslope fire spread, and their interactions. Grove-level random intercepts absorbed broad-scale spatial dependence and unobserved heterogeneity (e.g., fire weather, elevation), supporting causal inference by leveraging within-grove variability in the fixed effects to estimate treatment and structural effects while adjusting for observed confounders (see causal diagram in Supplementary Fig. 3). When evaluated against the independent field-validated sequoia mortality observations available within each subset (median = 233 trees), the GLMM ensemble showed good discrimination, with a median AUC of 0.82 across 500 ensemble draws (95% CI: 0.72–0.90; Supplementary Methods).

**Fig. 3:**
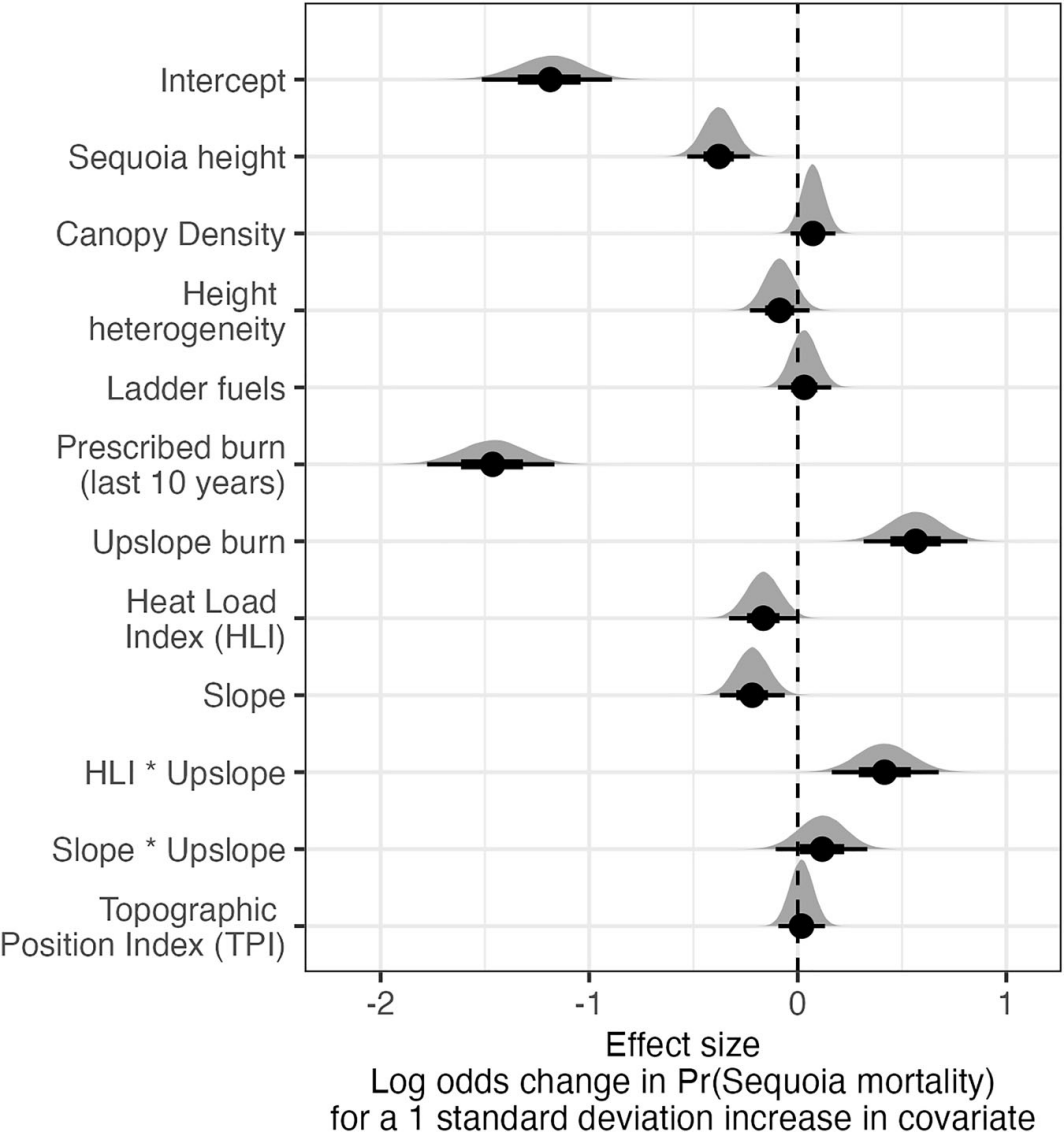
Posterior effect estimates of prescribed burning, forest structure, and topographic covariates on giant sequoia mortality. Posterior distributions from a Bayesian logistic regression model ensemble estimating giant sequoia mortality probability given prescribed burn history, forest structure characteristics, topography, and grove-level random effects. Distributions were pooled across 500 spatially balanced generalized linear mixed models fit to Bernoulli mortality realizations, with each model independently fit to a spatially balanced randomized subset (*n* = 3501 trees) sampled from 26,403 giant sequoias across 19 groves. Gray shaded areas represent posterior probability densities. Black markers represent posterior medians, with thick and thin intervals denoting 66% and 95% Bayesian credible intervals, respectively. Grove-level random effects are presented in Supplementary Fig. 4. Source data are provided as a Source Data file.

GLMM ensemble estimates revealed a strong effect of prescribed burning and several weaker or variable effects among structural and environmental covariates (Fig. 3). Recent prescribed burning had a large negative effect on mortality (*β*_prescribed burn_ = −1.47; 95% CI: [−1.78, −1.17]), corresponding to a 77% reduction in mortality odds (95% CI: [69%, 83%]). Of the lidar-derived structural metrics, ladder fuels, height heterogeneity and canopy density showed no clear effects on mortality, with 95% Bayesian credible intervals overlapping zero (Fig. 3). Other covariates revealed additional landscape-mortality relationships: taller sequoias were more likely to survive (*β*_*height*_ = −0.38; 95% CI: [ −0.53, −0.23]), upslope burns increased mortality risk (*β*_upslope_ = 0.57; 95% CI: [0.31, 0.81]), and that risk was amplified under high solar exposure (*β*_HLI×upslope_ = 0.42; 95% CI: [0.16, 0.68]).

### Counterfactual prescribed burn scenarios

**Fig. 4:**
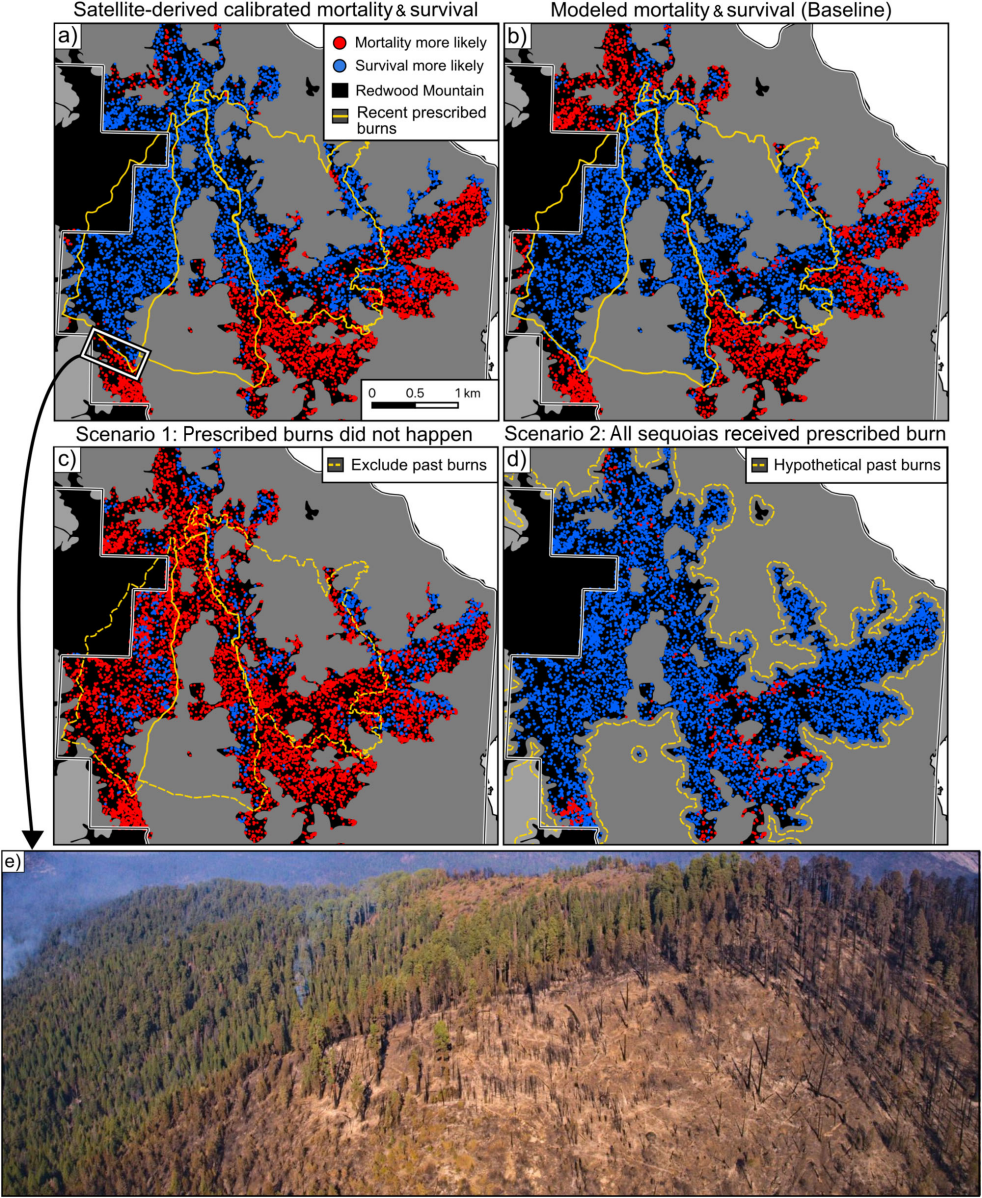
Observed and counterfactual giant sequoia mortality scenarios at Redwood Mountain grove. Case study at Redwood Mountain grove, burned in the KNP Complex (2021). Individual sequoias are colored red or blue if mortality was more likely (*p* > 0.5) or less likely (*p* < 0.5) based on posterior mean mortality probability. Shown are **a** calibrated satellite-derived outputs, **b** simulated mortality probabilities under the baseline scenario, **c** simulated mortality without prescribed burns, and **d** simulated mortality if all trees were treated with prescribed burns. Solid yellow lines represent prescribed burns within the 10 years preceding the KNP Complex wildfire, while dashed yellow lines represent counterfactual prescribed burn scenarios. The white boundary with dark gray area represents the national park boundaries. Also shown is **e** an aerial photograph showing (foreground) high sequoia mortality in an area that had not experienced fire in many decades, and (background) high sequoia survival in an area treated with prescribed fire in 2012 (image by Anthony Caprio [NPS] of the southwest corner of the grove). Includes copyrighted material of Planet Labs PBC. All rights reserved.

Using posterior draws from the GLMM ensemble, we simulated giant sequoia mortality under two counterfactual management scenarios, conditional on observed fire weather and landscape context. Under observed treatments (baseline), median mortality was 8,188 sequoias (95% CI: 7516–8958), falling within the uncertainty of the Step 1 estimate. In the no-treatment scenario (i.e., assuming no prescribed burns in the prior decade), mortality rose to 10,085 (95% CI: 9332–10,910), implying that existing treatments saved approximately 1900 sequoias. Conversely, if all sequoias had been subject to prescribed burning, mortality fell to 4300 (95% CI: 3543–5125), meaning an additional ~3900 sequoias would have survived in the simulated treatment scenario. The largest swing occurred at Redwood Mountain grove, with estimated deaths of 4580 (95% CI: 4219–4955) under baseline conditions, rising to 6412 without treatment (95% CI: 5908–6916), and falling to 3065 (95% CI: 2601–3547) under full treatment (Fig. 4).

### Sensitivity analysis

We evaluated the robustness of the GLMM ensemble inferences by modifying how prescribed burning and forest structure were defined and included in the models. First, expanding the prescribed burn window from 10 to 15 years increased the treated fraction from 22.5% (5945 trees; 3/19 groves) to 33.3% (8789 trees; 5/19 groves) but produced a similar effect size (Supplementary Figs. 5, 6). We then added a second indicator for burns 10–20 years before the KNP and Castle fires (a category that also included wildfires), which raised coverage to 39.5% (10,445 trees; 6/19 groves): the ≤10 year burns had a strong effect in reducing mortality risk (85% odds reduction; 95% CI: 75–90%), whereas 10–20-year burns still reduced odds by 25% (95% CI: 15–30%) (Supplementary Fig. 7).

To test for potential confounding or mediation, we reran models without forest structural variables and found that prescribed burn effects remained stable, indicating lidar metrics did not bias estimates, which is important given lidar was collected after prescribed burns (Supplementary Fig. 8). Further checks including removing the burn term (Supplementary Fig. 9), excluding trees that were treated by prescribed burns (Supplementary Fig. 10), and varying the spatial neighborhood size of structure metrics (Supplementary Fig. 11) revealed no significant changes in effects or model fit (LOOIC), underscoring the robustness of treatment estimates.

## Discussion

Our landscape-scale, tree-level analysis of ~26,000 giant sequoias reveals that prescribed burns within the preceding decade reduced wildfire mortality odds by approximately 77% (95% CI: 69–83%), with treated sequoias nearly four times more likely to survive (Fig. 3). Counterfactual simulations suggest existing treatments prevented ~1900 deaths across Sequoia and Kings Canyon national parks, and universal treatment could have saved an additional ~3900 trees. These estimates were derived from a sequoia-calibrated deep learning model embedded in a Bayesian framework that propagates classification and model uncertainty. By comparing treated and untreated trees within groves and adjusting for topography and grove-level heterogeneity, we find a substantial treatment effect of prescribed fire. In contrast, neighborhood forest structure metrics showed no significant or consistent associations with mortality, underscoring the importance of recent burning for survival at the tree level. Together, these findings show how tree-level remote sensing can deliver species-specific insights, providing a framework for other fire-threatened ecosystems.

More broadly, previous prescribed burns likely contributed to the survival of more sequoias than the  ~1900 estimated here. Our counterfactual simulations quantify survival benefits only within areas that ultimately burned, and do not account for potential effects of treatments on wildfire spread, suppression opportunities, or final fire perimeters. By reducing fuels, lowering fire intensity, and improving conditions for fire crews, past burns likely facilitated the successful containment of the KNP Complex wildfire soon after it entered the Giant Forest and Atwell groves (Supplementary Fig. 5). Without these treatments, containment would likely have been less effective, more grove area would have burned, and additional sequoias would have been lost.

### Prescribed burn effects

For millennia, giant sequoias persisted under frequent, lower severity surface fires that reduced fuels and promoted regeneration^13,36^. Twentieth-century fire exclusion disrupted this regime, allowing surface and canopy fuels to accumulate^10,14^. Prescribed burns, applied under moderate weather and fuel moisture conditions, reintroduce lower-severity fire to restore this ecological process^10,23^.

Our findings are consistent with surface fuels being a key driver of sequoia mortality risk: prescribed burning had a strong negative effect on mortality, while forest structure metrics showed little explanatory power (Fig. 3). This pattern indicates that the dominant effects of prescribed fire operate through surface and near-surface fuel attributes that are not fully captured by canopy-scale lidar metrics. The burn effect is likely mediated by reported mechanisms such as prolonged burning at the sequoia base, flame ascent through fire-scarred boles that conduct fire toward the crown and structurally weaken trees^1^, and crown scorch from convective heat^37^. Although previous studies have shown that thinning and burning reduce fire severity across Sierra Nevada forests^26^, our results provide species-specific evidence that prescribed fire substantially reduces mortality risk for giant sequoias, even under extreme wildfire conditions.

The prescribed burn effect remained stable across multiple sensitivity checks: expanding the recent treatment window from ≤10 to ≤15 years, removing structural covariates, or altering model structure all yielded nearly identical reductions in mortality odds (Supplementary Figs. 6, 11). Further, excluding canopy density, ladder fuels, and height heterogeneity showed minimal influence on the prescribed burn coefficient (Supplementary Fig. 8), indicating these structure metrics did not confound or mediate the effect. Adding a category for burns 10–20 years prior, which included past prescribed burns and wildfires, revealed a weaker but still significant effect. Mortality odds were reduced for older burns, though not as strongly as for more recent burns (Supplementary Fig. 7). Due to limited sample sizes for the older treatment category and the potential for conflation with wildfire effects, we treated this as a model check.

Much of our statistical power came from Redwood Mountain Grove, where a mosaic of prescribed burns (2011, 2012, 2016) and high wildfire mortality created strong within-grove contrasts (Fig. 4). This grove exemplifies how treatment history shapes survival outcomes, with stark differences between no-treatment and universal-treatment scenarios. While idealized, these counterfactual simulations illustrate the potentially large-scale effects of prescribed burning and demonstrate how tree-level inference can provide managers with evidence-based tools to anticipate the benefits of expanded treatments.

### Limited effects from neighborhood forest structure

Ecological theory and prior studies indicate that elevated ladder fuels promote vertical flame spread into tree crowns, while dense canopies facilitate fire spread within the canopy, and more heterogeneous stands with canopy gaps tend to resist severe fire^10,14,24,25,38^. We therefore expected lidar-derived structure metrics to help explain variation in giant sequoia mortality. However, these metrics consistently offered minimal explanatory power, a pattern that held across multiple sensitivity checks. Under extreme fire weather, variation in surface fuel loads may exert a dominant influence on fire behavior and subsequent mortality, whereas local variability in canopy structure provides limited additional discriminatory power at the individual-tree scale.

Our finding of minimal effects of forest structure on sequoia mortality should not be taken as evidence that restoration of canopy structure is unwarranted in sequoia groves. Several factors specific to our study may explain the limited structural effects. First, 2021—the year of the KNP Complex wildfire—was the hottest and driest in the >120-year record^39^, potentially creating extreme fire conditions that were not reflective of fires burning under less extreme conditions. For example, the southern part of Redwood Mountain grove burned as a plume-driven fire that created its own extreme winds. Second, topographic variables (e.g., slope, heat load, topographic position) that influence fire spread and moisture likely captured some of the underlying variation of forest structure, potentially masking structure-specific signals. Third, sequoia groves already exhibit high structural heterogeneity, possibly limiting our ability to detect benefits compared to more uniform second-growth forests^24^. Finally, our lidar-based ladder fuel metric, which measures mid-canopy return density within a fixed radius, may oversimplify the complex continuity of fuels that drives flame movement into the canopy^23,25^. Future studies that pair lidar with more nuanced fuel continuity metrics and with mechanical thinning experiments could better isolate the role of structure in moderating sequoia survival.

### A new approach to tree-level remote sensing

This study introduces a remote sensing framework that resolves wildfire outcomes at the scale of individual trees. By combining species-level stem maps, PlanetScope imagery, lidar, and field calibration within a Bayesian framework, we generate high-resolution mortality and survival estimates with explicit uncertainty. Analyzing thousands of trees across multiple groves allows us to incorporate random effects that account for background variation such as elevation and grove-level heterogeneity, thereby isolating treatment effects from broader environmental gradients. This tree-level resolution enables species-specific analyses that are difficult to achieve in mixed stands with coarser imagery, where survival and mortality of individual trees are often obscured; traditional fire severity assessments based on 30 m Landsat data may therefore mischaracterize species responses^26,30^.

The approach is transferable to other forest systems, with important considerations. Here, we integrated pre-existing stem maps with automated lidar-based crown delineation to enable precise tree-level mortality attribution and neighborhood structure calculation; where stem maps are unavailable, lidar alone can identify individual trees, though species identification becomes more challenging. Giant sequoia groves characterized by large crowns (often >10 m diameter) and open canopy structure are well suited to 3 m PlanetScope imagery, whereas denser forests with smaller crowns (<3 m) may require finer-resolution imagery or sub-pixel approaches. Despite these constraints, tree-level analyses enable species-specific inference that provides managers greater confidence in treatment effects. As old-growth trees increasingly face climate-exacerbated disturbance regimes, methods that resolve individual tree outcomes will be essential for guiding conservation strategies

Ongoing climatic changes have amplified the frequency and intensity of disturbances in old-growth forests, placing some of the world’s most iconic species at unprecedented risk. Giant sequoias, once considered highly resistant to fire, are now increasingly vulnerable to high severity wildfires. In this context, managers and policymakers need species-specific analyses to guide decisions about where and how to deploy fuel treatments. Our study demonstrates that fine-scale remote sensing can deliver this precision. By integrating species-specific stem maps, PlanetScope imagery, lidar, and field calibration within a Bayesian framework, we provide robust estimates of how prescribed burning influences survival. We show that recent prescribed burns substantially reduced sequoia mortality during the 2020–2021 wildfires and likely saved thousands of trees across Sequoia and Kings Canyon national parks. These findings identify prescribed fire as an effective and immediate tool for conserving giant sequoias. More broadly, they illustrate how high-resolution remote sensing enables species-specific conservation insights that are rarely attainable in large-scale studies and will be essential for safeguarding old-growth forests globally as disturbance regimes intensify.

## Methods

### Study area

This study was conducted in Sequoia and Kings Canyon national parks, specifically within the KNP Complex and Castle fire perimeters of the southern Sierra Nevada, California (Fig. 1). Our analysis focuses on the giant sequoia (*Sequoiadendron giganteum* L. Buchholz), which naturally exists in a limited range of ~97 sub-populations, or “groves,” along the western Sierra Nevada of California. The study groves are dominated by old-growth sequoia forest (but include second-growth stands), and span elevations from 1100 to 2400 m. The climate is Mediterranean, characterized by cool, wet winters and warm, dry summers, with roughly one half of annual precipitation falling as winter snow. The groves consist of mixed-conifer forests dominated by white fir (*Abies concolor*), sugar pine (*Pinus lambertiana*), incense cedar (*Calocedrus decurrens*), ponderosa pine (*P. ponderosa*), and red fir (*A. magnifica*).

Fire has been a dominant ecological force in giant sequoia groves for millennia^11,16^. Prior to Euro-American settlement, fire regimes were defined by frequent low severity burns within mosaics of small moderate and high severity patches^36^. This resulted in variable forest structure and composition, limited surface fuels, and fine-scale spatial heterogeneity, leading to greater resistance to disturbances including weather extremes, insect damage and wildfire^10,11,16^.

### Data

#### Giant sequoia stem locations and fire data

We used an existing spatial database of giant sequoia stem locations (the Sequoia Tree Inventory, STI) containing the point coordinates of almost all individual sequoias within Sequoia and Kings Canyon national parks, which were surveyed in the 1960s and 1970s, with coordinates later refined using airborne lidar and imaging surveys collected in 2015–2017. We selected STI tree locations within the Castle (2020, 70,828 ha) and KNP Complex (2021, 35,737 ha) wildfire perimeters, as defined by CALFIRE’s Fire and Resource Assessment Program (FRAP) database, and excluded trees within 30 m of the wildfire perimeter to avoid edge effects. We selected 19 groves that contained at least 30 giant sequoias each (Fig. 1). The number of sequoias per grove ranged from 36 to 10,236, with a mean of 1390 individuals. Using STI metadata, we further filtered the dataset to exclude giant sequoias observed as dead before the 2020–2021 wildfires, treated multi-stem clusters as individuals, included only trees >20 m in height according to the lidar-based canopy height model (CHM), and excluded observations with RA/RB or R1/R2 suffixes that represent regeneration polygons (not individual trees). This resulted in 26,403 unique trees for modeling objectives 1–3.

A subset of sequoias in the STI database (*n* = 1566) was visited post-fire, during which observers recorded mortality or survival status and estimated proportional crown scorch or torch effects (Supplementary Fig. 2). These field observations were distributed across 14 of 19 SEKI groves and included a balanced range of outcomes (753 surviving and 813 dead trees). This dataset was used for independent calibration and accuracy assessment of satellite-derived mortality and survival estimates (Supplementary Methods).

To characterize pre-fire forest structure, delineate tree-level sequoia crowns, and generate terrain-based covariates, we used discrete airborne lidar collected in August 2015 and July 2016 onboard the Carnegie Airborne Observatory aircraft, which collected on average 10.25 points/m^2^, and covered an area of 5783 ha in Sequoia and Kings Canyon national parks and adjacent areas^40^.

Tree-level giant sequoia mortality was derived from our published deep learning model trained and tested to classify PlanetScope 4-band time series into tree mortality or survival at 3 m spatial resolution^34^. This approach uses a spatio-temporal CNN calibrated with over one million trees labeled as mortality or survival from pre- and post-fire aerial images across 15 wildfires and four broad ecoregions in California. Here, each pixel is predicted as mortality if it lost >90% of its vertically projected photosynthetic crown foliage between a pre-fire year and a post-fire year^34^. This model uses spectral texture and pre- and post-fire monthly time series to predict the probability of each 3 m pixel into tree mortality or tree survival (as well as three additional classes for shrub survival, shrub mortality, or non-woody) using a softmax activation function. For the Sierra Nevada, the estimated user’s (precision) and producer’s accuracy (recall) ranged from 84.1–86.1% to 81.9–82.9%, respectively^34^. Using the trained model to predict at the Castle and KNP fires, we collected all PlanetScope imagery with <20% cloud cover and calculated median monthly mosaics from May to October for pre-fire (Castle = 2019, KNP = 2020) and post-fire years (Castle = 2021, KNP = 2022). The output tree mortality and survival probabilities were then calibrated (Supplementary Methods) using the previously mentioned field dataset of observed mortality.

Additional ancillary fire data were used to characterize historical prescribed burn perimeters and upslope burn conditions. Prescribed burn polygons were obtained from a spatial database containing historical fire in the national parks^19^. Fires burning upslope generally burn at higher intensity with higher flame lengths and faster spread rates^23^, so it is important to know whether trees on high slopes and/or high solar exposure experienced upslope or downslope fire. To categorize binary upslope conditions for each giant sequoia, we used daily fire progressions from a published model for the KNP and Castle fires using Visible Infrared Imaging Radiometer Suite (VIIRS) that estimates fire front perimeters at 24 h intervals^41^.

### Combining sequoia-level remote sensing datasets

We developed a workflow to integrate giant sequoia stem locations, pre-fire airborne lidar, and post-fire satellite-derived mortality data to estimate tree-level mortality probabilities for 26,403 individuals used in Questions 1–3. First, we generated crown footprint polygons for each sequoia, enabling spatial intersection with PlanetScope mortality/survival pixels and isolation of individual trees to quantify neighborhood forest structure from lidar. Crown delineation was performed using an unsupervised segmentation algorithm in the lidR package^42^. We created an interpolated digital terrain model (DTM) using K-Nearest Neighbors and inverse-distance weighting (IDW) with *K* = 10 nearest points and power 2 for IDW. Subtracting point heights from the DTM yielded a CHM, which was rasterized at 1 × 1 m resolution. Local maxima within a 6 m kernel identified treetops, from which convex crown polygons were generated to represent individual tree footprints^43^. Outputs of this segmentation process are shown in Fig. 2.

We intersected the lidar-derived crown polygons with the STI point locations to create a unique crown polygon for each giant sequoia. This produced a dataset with one-to-one matches and where multiple STI points intersected a single lidar-derived crown polygon; in these cases, we selected the STI point closest to the crown centroid, and we describe this dataset as “Quality 1”, which represented 20,251 giant sequoias (76.7%). For the remaining 6,152 trees, they had either no lidar crown polygon match (indicating their coordinates were slightly off relative to the lidar data, or the tree had fallen) or had more than one STI point within the intersecting crown polygon. For these cases, we created a 6 m buffer around the STI point as the crown polygon (“Quality 2”). Trees with more than 90% of their PlanetScope-mortality pixels classified as shrub or non-woody were also excluded from the analysis, as we assume their coordinates were incorrect relative to the satellite imagery and lidar (663 total trees excluded). Trees with Qualities 1 and 2 were used in summarizing giant sequoia mortality (Question 1) and scenarios (Question 3), while only Quality 1 trees were used in modeling prescribed burn and canopy structure effects (Question 2).

To generate species-specific mortality estimates for giant sequoias, the outputs of the CNN classifier from Dixon et al.^34^, originally trained across multiple tree species, were post-processed using an independent dataset of 1566 field-observed sequoias collected in 2022 and 2023. Here, the crown polygon for each sequoia was used to extract all intersecting 3 m pixels from the CNN model output, then assigned a median probability value for classes including tree mortality, tree survival, non-woody, and shrub. Each tree had a recorded post-fire survival status and fractional crown scorch/torch; for calibration purposes, any individual with scorch + torch volume exceeding 90% was labeled as dead^1^, resulting in 813 dead and 753 live observations.

A logistic calibration model was fit using the CNN-predicted mortality and survival probabilities as inputs; this step also helps correct for differences in probabilities from non-woody and shrub classes. Model performance was evaluated using five-fold cross-validation. The resulting posterior mortality probabilities contain uncertainty from the neural network and its species-specific adjustment to giant sequoias, and also formed the foundation for subsequent analyses, including total mortality estimation and modeling the effects of prescribed burning and forest structure. Calibration description and results are presented in Supplementary Methods.

### Bayesian workflow overview

To address Questions 1–3, we implemented a three-stage Bayesian workflow that combines calibrated tree-level mortality probabilities (*N* = 26,403) with mixed-effects modeling and counterfactual simulation (Supplementary Fig. 1).

In Stage 1, we drew binary mortality outcomes for each tree by sampling from its calibrated posterior mortality probability. We repeated this procedure 6000 times to generate 6000 imputed mortality datasets, which were summarized to estimate grove-level and park-wide mortality totals (Question 1).

In Stage 2, we fit an ensemble of logistic generalized linear mixed models (GLMMs) to a subset of these imputed datasets to estimate associations between mortality and prescribed burning, forest structure, and other covariates (Question 2). Each GLMM was fit to a spatially balanced subset of trees (*n* = 3501) and included a grove-level random intercept. Posterior samples were pooled across the ensemble to summarize effect sizes.

In Stage 3, we simulated two counterfactual prescribed-burn scenarios (universal treatment and no treatment within the prior decade) by modifying the prescribed-burn indicator and applying posterior draws from the Stage 2 ensemble to all trees. For each scenario, we generated predicted mortality probabilities and drew binary mortality outcomes with 6000 Bernoulli trials to estimate mortality counts (Question 3).

### Causal inference framework and model inputs

We used a causal inference analytical framework^44^ to estimate the effects of prescribed burn history and neighborhood forest structure (variables of interest) on giant sequoia mortality through their influence on fire behavior, which was not directly observed. A directed acyclic graph (DAG) representing hypothesized relationships among treatments, covariates, and mortality at the tree level is shown in Supplementary Fig. 3. Based on this DAG, we applied the backdoor criterion to identify an adjustment set that closes non-causal paths and allows a causal interpretation of estimated effects, conditional on the stated assumptions.

Prescribed burn exposure was represented at the tree level as a binary indicator denoting whether a focal sequoia existed within 30 m of a prescribed burn perimeter occurring within the 10 years preceding wildfire (Supplementary Fig. 5). The 30 m buffer accounts for spatial uncertainty in historical fire perimeter mapping and captures potential edge effects of treated areas.

Neighborhood forest structure was characterized using three complementary lidar-derived metrics (Fig. 2). Canopy density and canopy height heterogeneity were quantified within a 90 m radius surrounding the focal tree, including lidar returns within the focal crown polygon, to capture variation in vegetative material and vertical structure associated with stand age, canopy gaps and openings^27^. Ladder fuels were quantified within 10 m of the focal crown polygon, excluding returns within the crown polygon, and represent the density of mid-canopy material capable of facilitating vertical flame propagation. Height heterogeneity was calculated using the rumple index, canopy density as the proportion of lidar first returns over 4 m, and ladder fuels as the proportion of returns between 4 m and half the focal tree height^27,45^. Together, these metrics represent distinct structural dimensions with minimal collinearity and established links to forest resistance to disturbance^24,25,27,38^.

Additional variables that influence fire behavior and subsequent sequoia mortality include terrain, sequoia height, burn direction relative to slope, and grove-level factors (e.g., elevation, climate, fire weather, and management; Supplementary Figs. 12–13). To account for confounding, we adjusted for key terrain attributes that can influence both forest structure and fire behavior: slope, heat load index (HLI), and topographic position index (TPI).

HLI was calculated as mean potential solar irradiation (Wh/m^2^, June-October, daily) from a 3 m lidar-derived DEM using the GRASS GIS r.sun module. TPI was calculated as the difference in elevation (m) between each pixel and the surrounding landscape using a 500 m radius. For each giant sequoia, we summarized the mean slope and HLI within a 120 m radius and TPI within a 30 m radius, reflecting broader-scale controls on fire behavior (slope, solar exposure) versus more localized topographic position effects.

Additional covariates included focal tree height and upslope burn conditions. Taller and older sequoias are generally more resistant to fire disturbance^1^; accordingly, tree height was quantified as the 95th percentile of lidar returns within each sequoia crown.

Fire moving upslope is typically associated with higher intensity and faster spread rates^23,46^. To characterize upslope fire exposure, we combined 24-h fire progression perimeters^41^ with terrain aspect derived from the DEM (Supplementary Fig. 12). For each tree, we estimated the azimuth of fire spread using perimeters from the day before and after burning, refined by visual inspection. This direction was compared to mean terrain aspect within 120 m of the focal tree. If the ±60^∘^ buffers around the fire spread direction and terrain aspect did not overlap, the tree was classified as having experienced upslope fire (value = 1); otherwise, it was classified as downslope or lateral fire (value = 0). Given uncertainty in fire front position and timing at fine scales, upslope exposure was treated as a binary indicator rather than a continuous measure. Interaction terms between upslope exposure and both HLI and slope were included to capture terrain-dependent differences in fire intensity.

### Estimating effects of prescribed burns and forest structure

To quantify the effects of prescribed burning, forest structure, and additional covariates on giant sequoia mortality (Question 2), we fit an ensemble of Bayesian GLMMs. Consistent with the causal framework, grove-level factors such as fire weather and elevation were accounted for using grove-level random intercepts. From the 6000 binary mortality draws generated in Stage 1 via Bernoulli sampling, we randomly selected 500 imputed datasets for model fitting. Each imputation represented a plausible realization of tree-level post-fire outcomes and thus contributed to uncertainty in the estimated effects. To reduce spatial autocorrelation, we constructed spatially balanced random subsets of sequoias by overlaying a 90 × 90 m grid and randomly selecting one tree per cell (*n* = 3501, or 13.2% of all trees), with the selection independently re-randomized for each model fit. We included only trees with high spatial precision (“Quality 1”) in this modeling step. This approach yielded 500 spatially balanced GLMMs that collectively captured uncertainty arising from satellite, sampling, and spatial heterogeneity. Posterior samples were then pooled across all 500 models to generate full distributions of covariate effect estimates. Model discrimination was evaluated post hoc using area under the receiver operating characteristic curve (AUC) calculated against independent field observations within each ensemble subset (Supplementary Methods).

For Eq. 1, let *y* be the response variable (tree mortality), where the model is specified as:

1 $${y}_{i} \sim 	 {{{\rm{Bernoulli}}}}({\phi }_{i})\\ {{{\rm{logit}}}}({\phi }_{i})=	{\beta }_{0}+{\beta }_{1}\,heigh{t}_{i}\\ 	+{\beta }_{2}\,ladder\_fuel{s}_{i}\\ 	+{\beta }_{3}\,heterogeneit{y}_{i}\\ 	+{\beta }_{4}\,canopy\_densit{y}_{i}\\ 	+{\beta }_{5}\,prescribed\_bur{n}_{i}\\ 	+{\beta }_{6}\,upslop{e}_{i}\\ 	+{\beta }_{7}\,TP{I}_{i}\\ 	+{\beta }_{8}\,HL{I}_{i}\\ 	+{\beta }_{9}\,slop{e}_{i}\\ 	+{\beta }_{10}\,(upslop{e}_{i}\times HL{I}_{i})\\ 	+{\beta }_{11}\,(upslop{e}_{i}\times slop{e}_{i})\\ 	+{\gamma }_{g(i)},$$

where *i* is the index of individual trees (*i* = 1, 2, 3, ...3501) and *g* is the index of grove (*g* = 1, 2, ...19), with *γ* capturing residual variation related to each grove. We used a Bernoulli family distribution with a logit link function. Continuous predictors (excluding binary variables) were centered and scaled before fitting the model to facilitate prior assignment and exploration of the posterior distribution by the sampler. And to adjust for identifiability of the intercept and random effect, we incorporated post-sweeping^47,48^, which re-centers the varying intercepts and the global intercept to remove potential collinearity between them.

The model was run in R^49^ using JAGS^50^ with weakly informative priors and three chains. We allowed 2,000 adaptation iterations to tune the samplers, followed by 1,000 burn-in iterations to ensure the chains converged before sampling. After burn-in, we collected 200 posterior samples per chain, checked chain mixing autocorrelations visually with trace plots, and ensured model convergence with Rhats <1.01. We fit the model across 500 imputations, each based on a unique Bernoulli draw from the calibrated mortality probabilities. This yielded 600 posterior samples per imputation (200 samples × 3 chains), resulting in a total of 300,000 posterior draws. We then summarized pooled posterior distributions using mean values with 66% and 95% credible intervals.

### Robustness and sensitivity analyses

We conducted a series of robustness and sensitivity analyses to evaluate the stability of our results to key modeling assumptions, including treatment definition, covariate inclusion, and spatial scale. Sensitivity to prescribed fire history was assessed using three separate model fits. First, we redefined the prescribed burn indicator by expanding the treatment window from ≤10 to ≤15 years prior to wildfire, increasing the proportion of treated trees from 22.5% (5945 trees; 3/19 groves) to 33.3% (8789 trees; 5/19 groves). Second, we added an additional indicator for burns occurring 10–20 years prior to wildfire (in addition to the original ≤10-year indicator), increasing total coverage to 39.5% (10,445 trees; 6/19 groves) to examine potential lagged treatment effects; because this longer window included both prescribed burns and wildfires, it was retained only as a diagnostic model check. Third, we fit a model excluding all forest structure predictors to evaluate whether estimated effects of prescribed burning were mediated or confounded by forest structure.

Additional analyses evaluated the robustness of forest structure effects. We assessed sensitivity to neighborhood size by re-running the primary model across 16 combinations of buffer radii (30, 60, 90, and 120 m) for canopy density and canopy height heterogeneity. Models were compared using leave-one-out information criterion (LOOIC) and pooled posterior effect sizes. We also fit a model excluding the prescribed burning variable entirely, as well as a model excluding all treated trees, to evaluate whether prescribed burns masked or altered estimated forest structure effects.

Together, these analyses demonstrate that our primary findings regarding prescribed burning and forest structure are robust across plausible alternative specifications and modeling assumptions. Detailed results, figures, and LOOIC comparisons are provided in the Supplementary Materials.

### Treatment scenarios and case study

To illustrate the magnitude of effects estimated in Eq. 1 (Question 2), we simulated two counterfactual management scenarios for all 26,403 trees (Question 3): (1) no prescribed burning, and (2) universal prescribed burning. We randomly sampled 6000 posterior draws from the primary GLMM pooled ensemble and re-evaluated the treatment variable under each scenario, setting the prescribed burn indicator to either 0 (untreated) or 1 (treated) for all trees. Using the corresponding posterior coefficients, we generated new predicted mortality probabilities for each tree under each scenario, then performed Bernoulli trials to estimate total mortality outcomes. Median values and 95% credible intervals were computed to obtain the mortality count under each scenario.

To spatially contextualize the results, we visualized these scenario outcomes at Redwood Mountain grove, the largest grove in our study, located within the KNP Complex fire perimeter. This grove contained 10,236 giant sequoias over 20 m in height, of which 5547 (54.2%) had been treated with prescribed fire in the previous decade.

### Reporting summary

Further information on research design is available in the Nature Portfolio Reporting Summary linked to this article.

## Supplementary information

Supplementary Information

Reporting Summary

Transparent Peer Review file

## Source data

Source Data

## Data Availability

Datasets required to reproduce the analyses presented in this study are publicly archived through Open Science Framework at 10.17605/OSF.IO/83S2U. Raw PlanetScope imagery is available under license from Planet Labs and is therefore not publicly redistributed. Source data are provided with this paper.

## Code availability

Code supporting the analyses, statistical modeling, scenario simulations, and figure generation presented in this study is publicly available through GitHub and archived through Zenodo at 10.5281/zenodo.20143683.
