# Supplementary material for: Previous prescribed burns saved thousands of ancient sequoias during historically unprecedented wildfires
Source: Nat Commun. 2026 Jul 11;17:8558. doi: 10.1038/s41467-026-75418-6 (PMC13483421; doi:10.1038/s41467-026-75418-6)
Supplement: Supplementary file 1 [file 41467_2026_75418_MOESM1_ESM.pdf]

Supplementary Information

Previous prescribed burns saved thousands of ancient sequoias during historically unprecedented wildfires

Dan J. Dixon, Adrian J. Das, Xiaoli Dong, Andrew M. Latimer,
David N. Soderberg, Nathan L. Stephenson, Anthony C. Caprio, Yufang Jin

This file contains Supplementary Methods, Supplementary Figures 1–16, and Supplementary Table 1.

Supplementary Methods

Bayesian workflow and uncertainty propagation

Here we provide full technical details of the three-stage Bayesian workflow summarized in the main text, including uncertainty propagation, model specification, and associated diagnostics. This three-stage process preserved uncertainty from satellite-derived tree mortality classification, spatial sampling, and model estimation through to inference and prediction.

In the first stage, we used Monte Carlo sampling to draw binary mortality outcomes from the calibrated posterior probabilities for each of the 26,403 giant sequoias. Over 6,000 draws, each imputed dataset represents a plausible realization of post-fire outcomes based on calibrated probabilities that fully account for their uncertainty (captured in the posterior distribution of each calibrated probability). To address Question 1, we report the total predicted sequoia median mortality counts with 95% Bayesian credible interval (CI) summarizing the 6,000 Bernoulli trials separated by grove.

Next, we fit an ensemble of logistic generalized linear mixed models (GLMMs) to each imputed dataset to estimate the effects of prescribed fire, forest structure, and other covariates on sequoia mortality. Each model incorporated spatially balanced random subsets of sequoias (n = 3,501 trees) and included grove-level random intercepts, which also account for unmeasured heterogeneity. Pooling posterior draws across all models yielded fully propagated estimates of effect sizes,

incorporating both classification and sampling uncertainty.

For Question 3, we simulated two counterfactual management scenarios for all 26,403 trees: (1) no prescribed burning, and (2) universal prescribed burning. We randomly sampled 6,000 posterior draws from the primary GLMM pooled ensemble and re-evaluated the treatment variable under each scenario, setting the prescribed burn indicator to either 0 (untreated) or 1 (treated) for all trees. Using the corresponding posterior coefficients, we generated new predicted mortality probabilities for each tree under each scenario, then performed Bernoulli trials to estimate total mortality outcomes. This step consisted of 6,000 Bernoulli simulations from the GLMM posterior distributions. Although these scenarios are not intended to reflect realistic management feasibility, they serve to illustrate the potential large-scale impact of prescribed burning on giant sequoia survival.

Tree-level mortality calibration

Model specification

The satellite mortality model used in this study is a previously published spatio-temporal convolutional neural network (ST-CNN) designed to detect canopy-scale tree and shrub mortality from PlanetScope imagery at 3-m resolution Dixon2023SatelliteLearning. The model jointly exploits spatial context and pre- and post-disturbance temporal dynamics using monthly PlanetScope composites, allowing it to distinguish heterogeneous patterns of survival and mortality at the scale of individual canopies. Training labels were generated using a semi-automated workflow that combines pre-fire airborne lidar crown segmentation with post-fire interpretation of very high-resolution aerial imagery, producing over one million labeled tree and shrub canopies across multiple California wildfires. The network was implemented in Python using TensorFlow/Keras and outputs class probabilities for tree mortality, tree survival, shrub mortality, shrub survival, and non-woody cover. In this study, we leverage the published model outputs and calibrate them specifically for giant sequoias to improve probabilistic accuracy and enable full uncertainty propagation through subsequent statistical analyses.

We used the ST-CNN outputs—probability of mortality (z_m) and survival (z_s)—as predictors in a logistic calibration model:

$$\text{logit}(\theta_i) = \alpha + \gamma_m z_{m,i} + \gamma_s z_{s,i} \tag{1}$$

where θ_i is the calibrated mortality probability for tree i. We used weakly informative priors: $\alpha \sim \mathcal{N}(0, 10^2)$, $\gamma_m \sim \mathcal{N}(0, 10^2)$, and $\gamma_s \sim \mathcal{N}(0, 10^2)$. The model was implemented in JAGS with 3 chains, 5,000 adaptation steps, 5,000 burn-in, and 2,000 posterior draws per chain.

Calibration model performance

All performance metrics reported in this section (F1 score, Brier score, recall, precision) evaluate the calibration model applied to the independent field dataset and do not assess the downstream GLMM ensemble. Posterior densities for all parameters (Supplementary Fig. 14) showed good mixing, indicating convergence and identifiability. Posterior estimates were: α: Mean = -0.86 (95% CI: -1.16, -0.58); γ_m: Mean = 0.68 (95% CI: 0.36, 1.03); γ_s: Mean = -2.49 (95% CI: -2.96, -2.07). Model performance was assessed using five-fold cross-validation using the independent dataset of 1,566 labeled sequoias (813 dead). Calibration improved probabilistic accuracy, with the mean Brier score decreasing from 0.095 to 0.079. Classification performance also improved: F1 score increased from 0.813 to 0.844, and recall rose from 0.751 to 0.845, with a modest tradeoff in precision (decreasing from 0.888 to 0.844).

Comparing uncalibrated and mean calibrated probabilities, calibration tended to shift values from overly confident to more conservative (closer to 0.5) (Supplementary Fig. 15). This was most noticeable for values < 0.25, indicating the satellite-based uncalibrated probabilities were insensitive to some cases of true mortality, which is also reflected in the increase in recall for calibrated values. Predictive posterior distributions for nine randomly selected trees (Supplementary Fig. 16) illustrate how calibration shifted or generated uncertainty around mortality probabilities, highlighting its role in aligning model outputs with sequoia-specific mortality.

Calibrated posterior draws θ_i were then sampled via Bernoulli trials to generate binary mortality outcomes for each tree, allowing uncertainty from the classification step to propagate into effect estimation and scenario modeling.

Causal inference framework

We used a causal inference analytical framework to estimate the effects of prescribed burn history and neighborhood forest structure ('variables of interest') on giant sequoia mortality via their manipulation of fire behavior (fire behavior not directly observed). A causal diagram of the studied system at the tree-level using a directed acyclic graph (DAG) is shown in Supplementary Fig. 3. Here, we estimate the causal effects of our variables of interest on tree mortality through the

unobserved mediator fire behavior. We applied the 'backdoor criterion' to the DAG to close non-causal paths and allow a causal interpretation of the slope of the variables of interest (conditional on the DAG's assumptions).

GLMM ensemble discriminatory performance

To assess the discriminatory performance of the GLMM ensemble independently of the calibration phase, we evaluated model predictions against field-validated sequoia mortality observations that were present within each spatially balanced modeling subset. For each of the 500 ensemble iterations, posterior-mean mortality probabilities were computed for all trees in the fitted subset, and the area under the receiver operating characteristic curve (AUC) was calculated using only trees with independent field-based mortality labels.

Because spatial subsampling varied across iterations, the number of labeled trees contributing to AUC estimation differed among ensemble draws (median = 233 trees). We summarize model discrimination using the median and 95% interval of AUC values across all ensemble iterations. This evaluation assesses the ability of the fitted models to distinguish mortality from survival among ground-observed trees and does not influence model fitting or counterfactual simulations.

Supplementary Figures

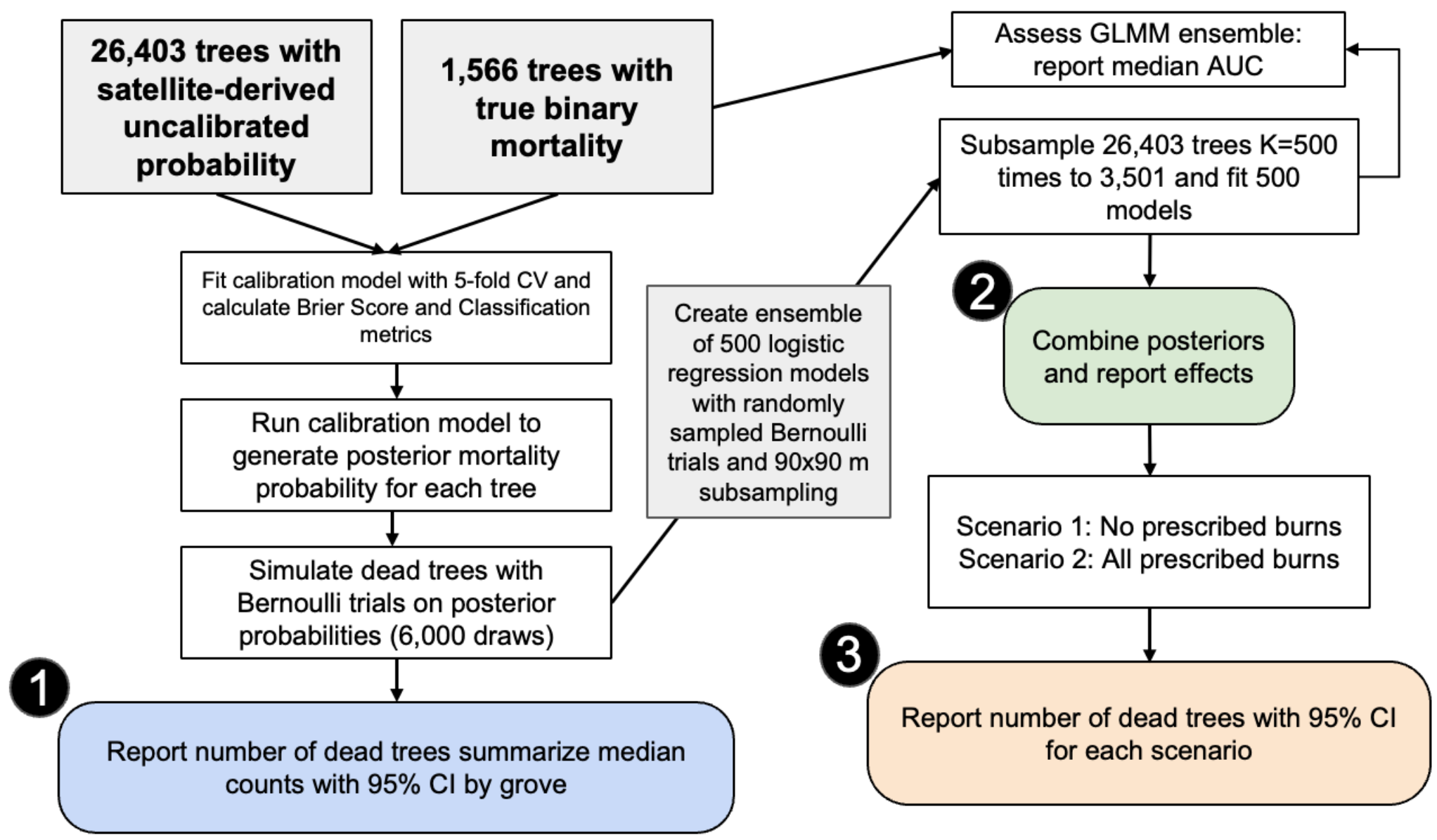

Supplementary Figure 1: Bayesian workflow diagram showing the three-stage uncertainty propagation used in this study: (1) sampling tree-level mortality outcomes from calibrated posterior probabilities, (2) fitting the GLMM ensemble to estimate covariate effects, and (3) generating counterfactual predictions under alternative prescribed-burn scenarios.

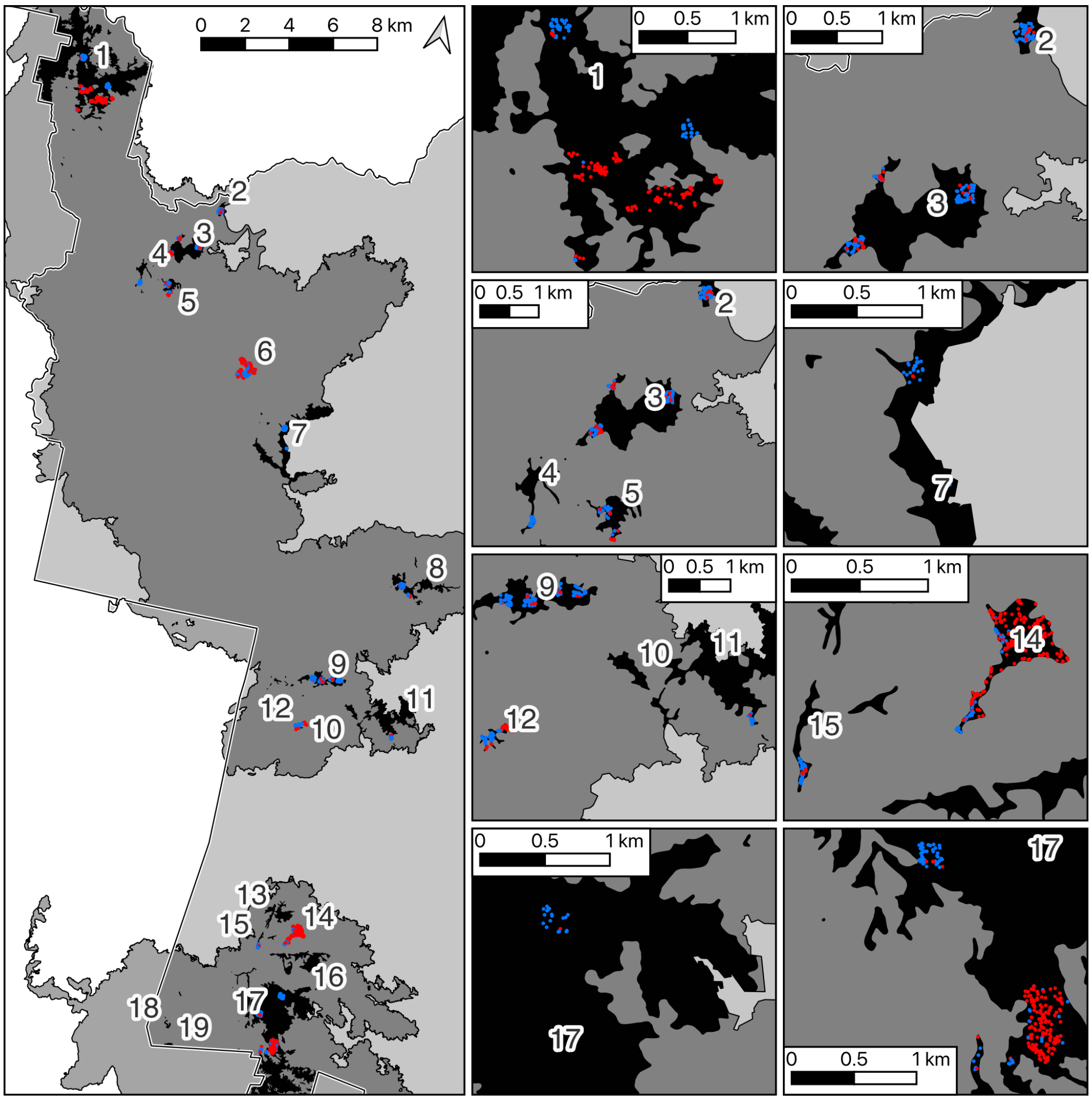

Supplementary Figure 2: Locations of post-fire field observations used for calibration and validation (red = dead, blue = survived) within study groves in Sequoia and Kings Canyon national parks and the KNP Complex and Castle fire perimeters. Observations were remapped using a 90% crown foliage loss threshold (red = dead, blue = survived).

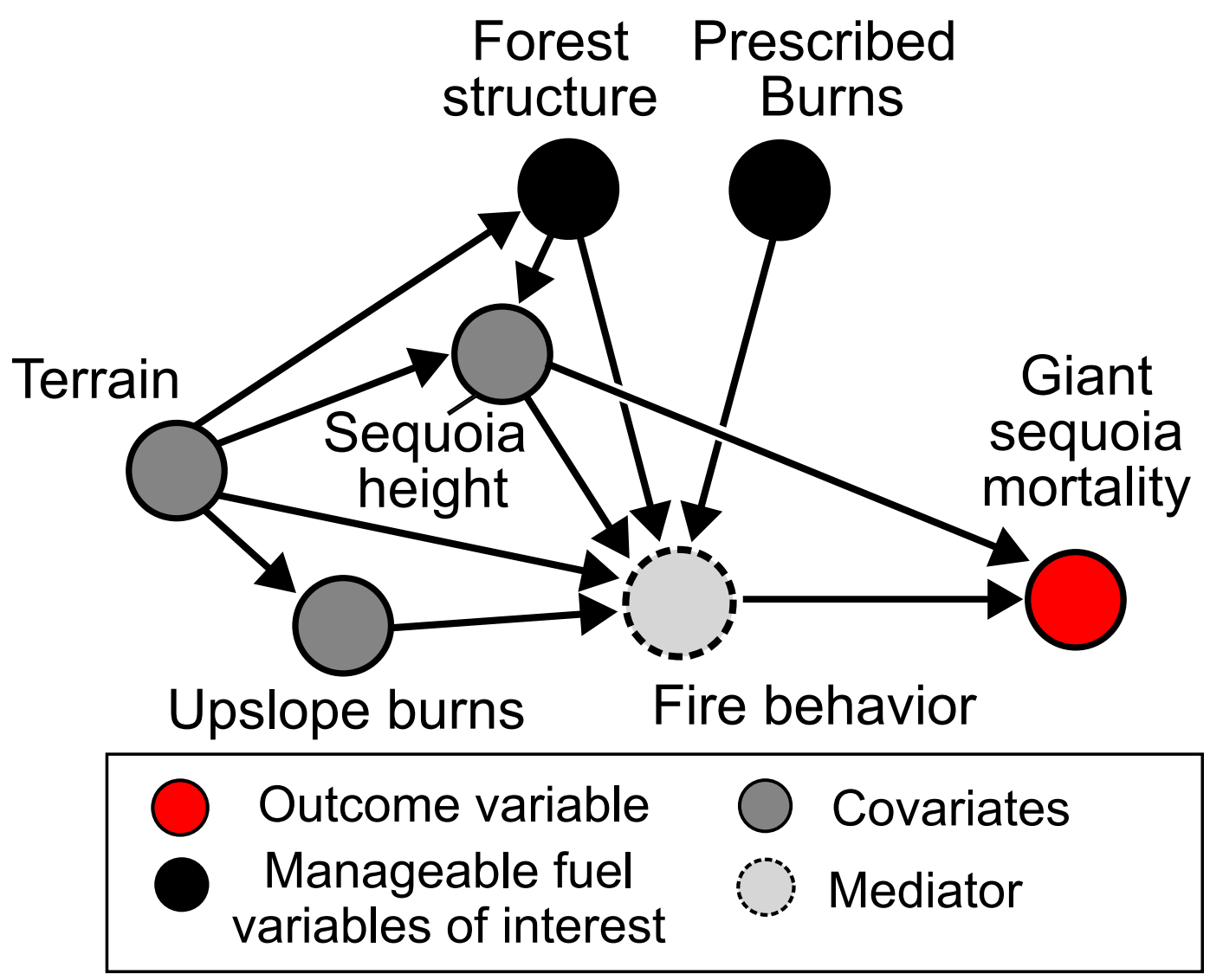

Supplementary Figure 3: Directed acyclic graph (DAG) illustrating hypothesized relationships among manageable fuel variables (black), covariates (gray), an unobserved mediator (fire behavior; dashed outline), and the outcome (giant sequoia mortality; red). Solid arrows denote hypothesized causal relationships used to define the adjustment set for estimating treatment effects. Forest structure was quantified after many prescribed burns and may reflect post-treatment conditions; we therefore evaluate sensitivity to potential mediation or post-treatment adjustment by repeating analyses without forest structure covariates. Grove-level factors such as fire weather and elevation, though not shown, are accounted for using grove-level random intercepts in the statistical models.

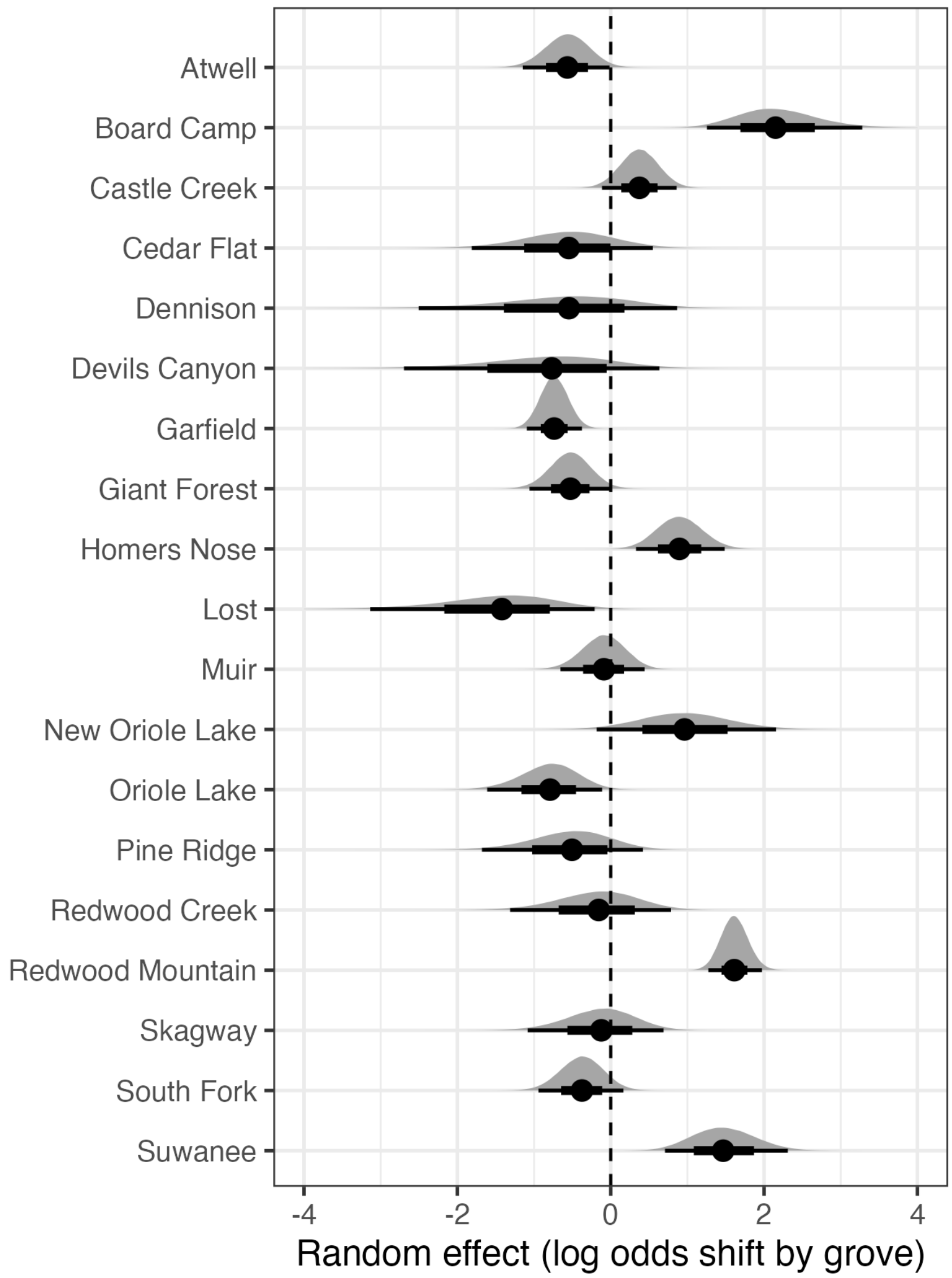

Supplementary Figure 4: Estimated grove-level random effects from the primary model. Each point represents the posterior distribution of the log-odds shift in mortality for one of 19 groves, capturing unmeasured variation across spatial units. Black markers represent the median of each distribution, bold and thin intervals surrounding the point estimate represent the 66% and 95% credible intervals, respectively. Source data are provided as a Source Data file.

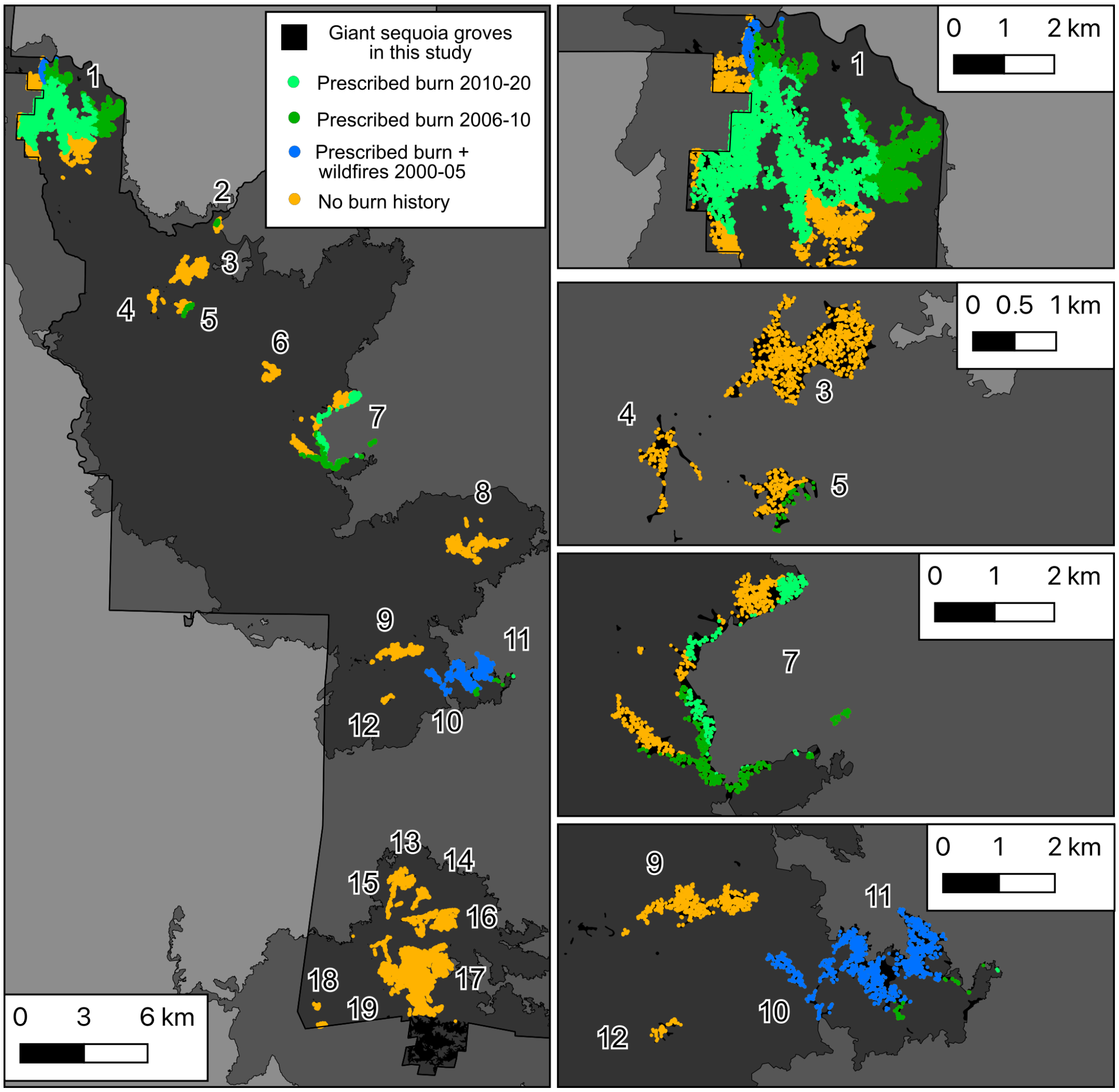

Supplementary Figure 5: Location and burn history of individual giant sequoias across 19 groves within Sequoia and Kings Canyon National Parks, prior to the KNP Complex and Castle fires. Each tree is colored by time since last wildfire: 0–10 years, 11–15 years, 16–20 years, or no recorded fire within 20 years. The 16–20 year category includes fires that partially intersected grove boundaries. Grove names and ID numbers correspond to Fig. 1 in the main text.

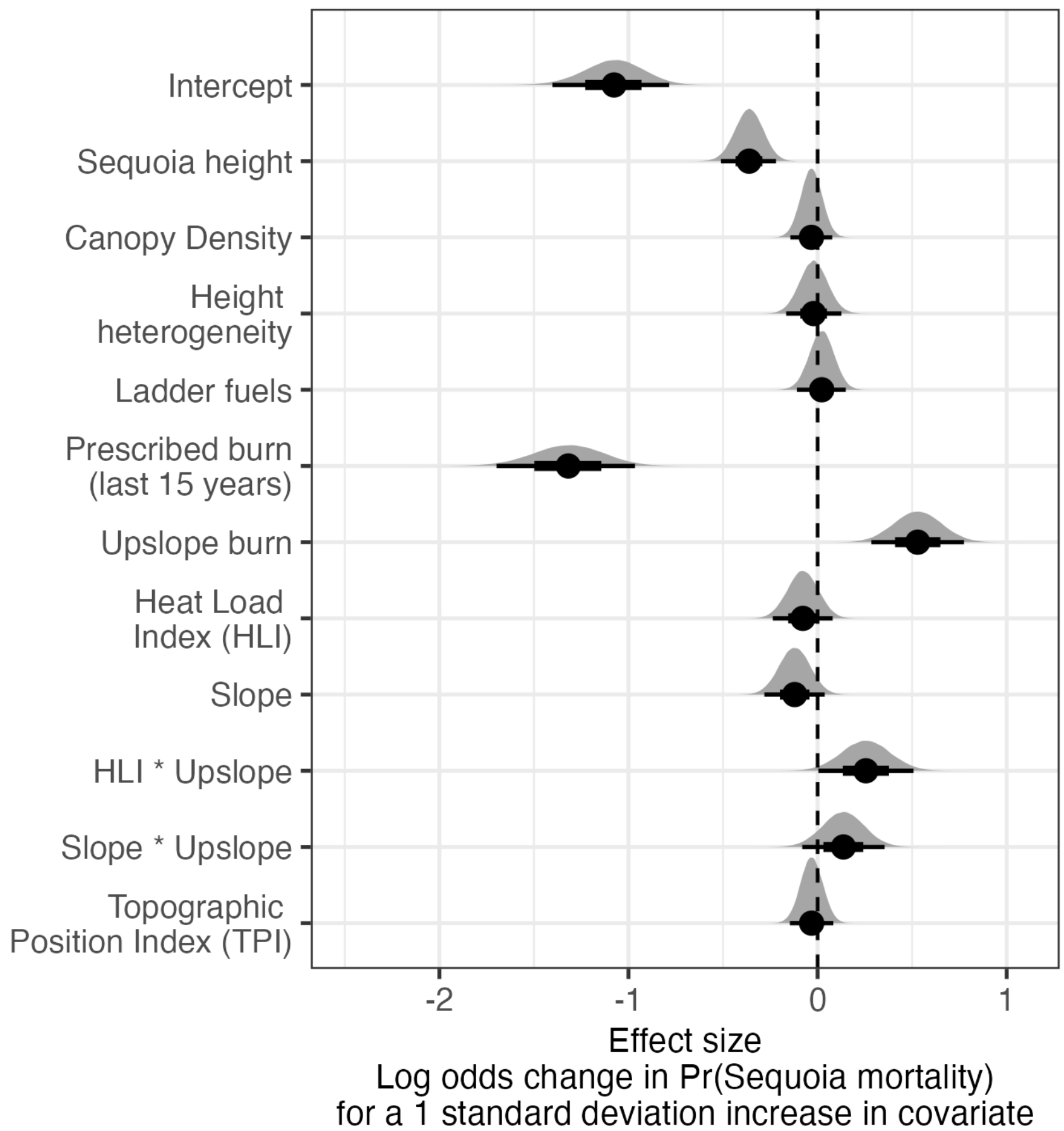

Supplementary Figure 6: Sensitivity check expanding the prescribed burning window from 10 to 15 years. Black markers represent the median of each distribution, bold and thin intervals surrounding the point estimate represent the 66% and 95% credible intervals, respectively. Source data are provided as a Source Data file.

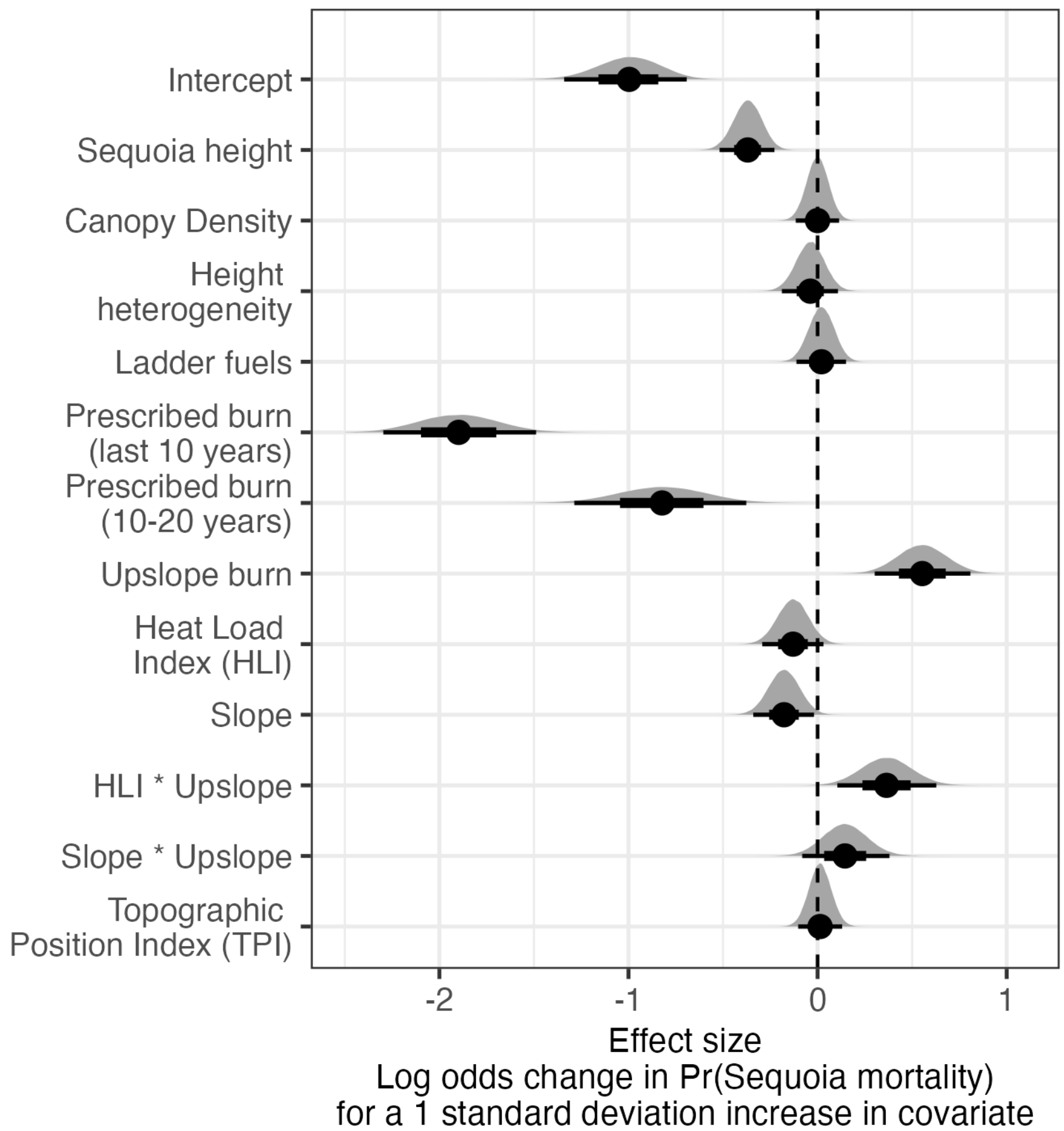

Supplementary Figure 7: Sensitivity check including a second prescribed burn indicator for burns conducted 10–20 years prior. Black markers represent the median of each distribution, bold and thin intervals surrounding the point estimate represent the 66% and 95% credible intervals, respectively. Source data are provided as a Source Data file.

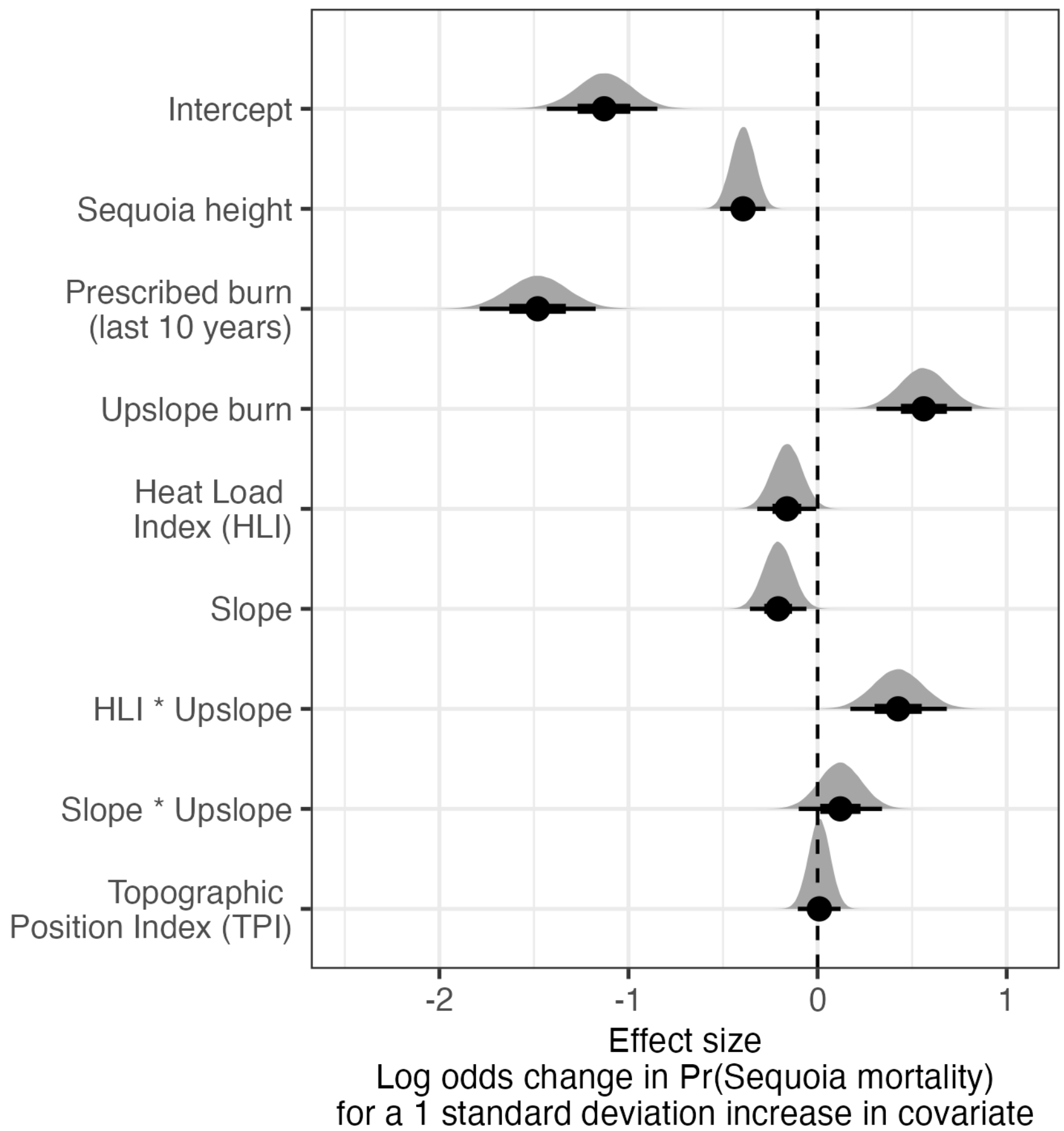

Supplementary Figure 8: Sensitivity check excluding all forest structure variables (height heterogeneity, ladder fuels, canopy density). Black markers represent the median of each distribution, bold and thin intervals surrounding the point estimate represent the 66% and 95% credible intervals, respectively. Source data are provided as a Source Data file.

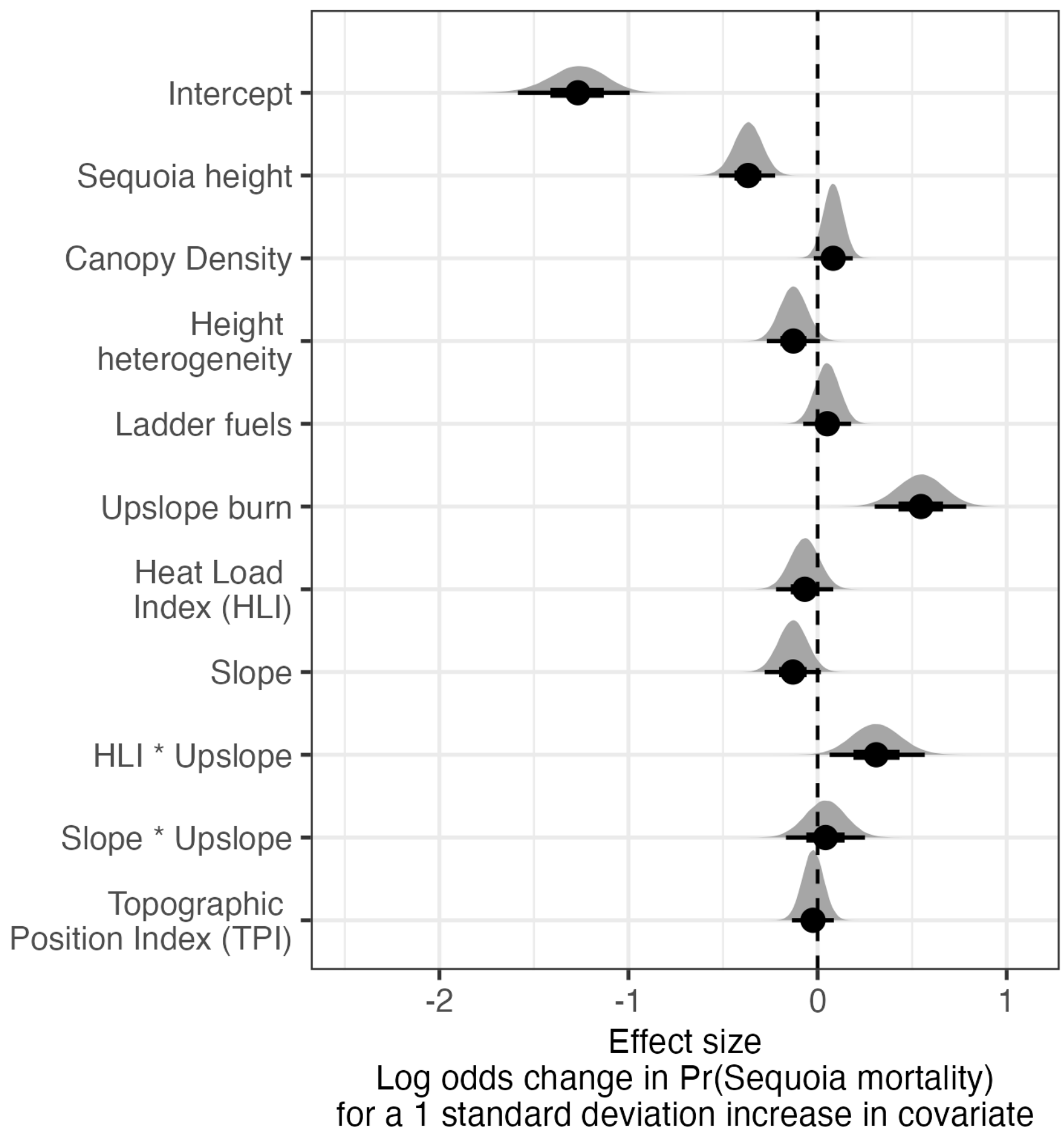

Supplementary Figure 9: Sensitivity check removing the prescribed burn variable. Black markers represent the median of each distribution, bold and thin intervals surrounding the point estimate represent the 66% and 95% credible intervals, respectively. Source data are provided as a Source Data file.

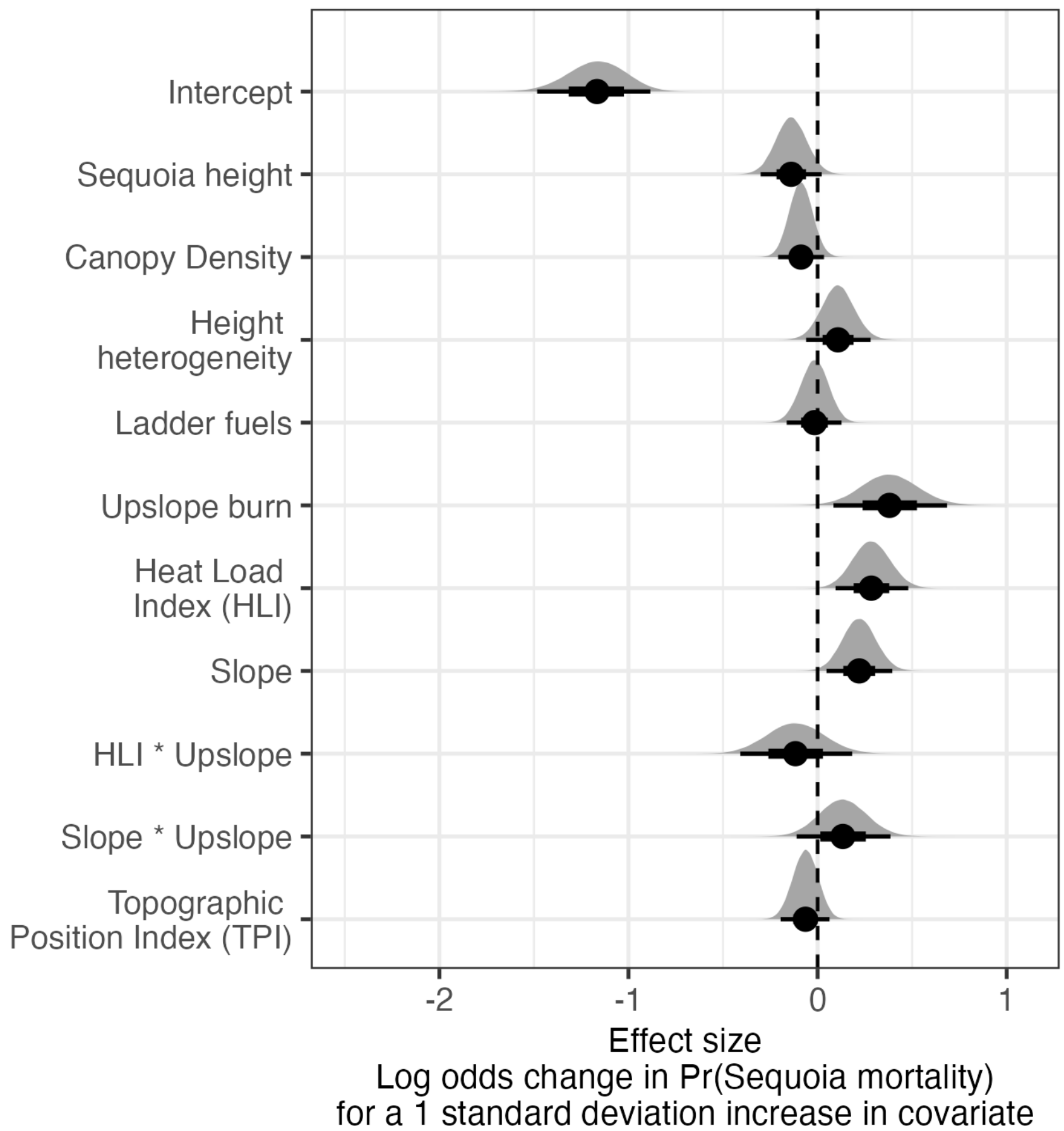

Supplementary Figure 10: Sensitivity check excluding all trees with recent prescribed burns and refitting the primary model. Black markers represent the median of each distribution, bold and thin intervals surrounding the point estimate represent the 66% and 95% credible intervals, respectively. Source data are provided as a Source Data file.

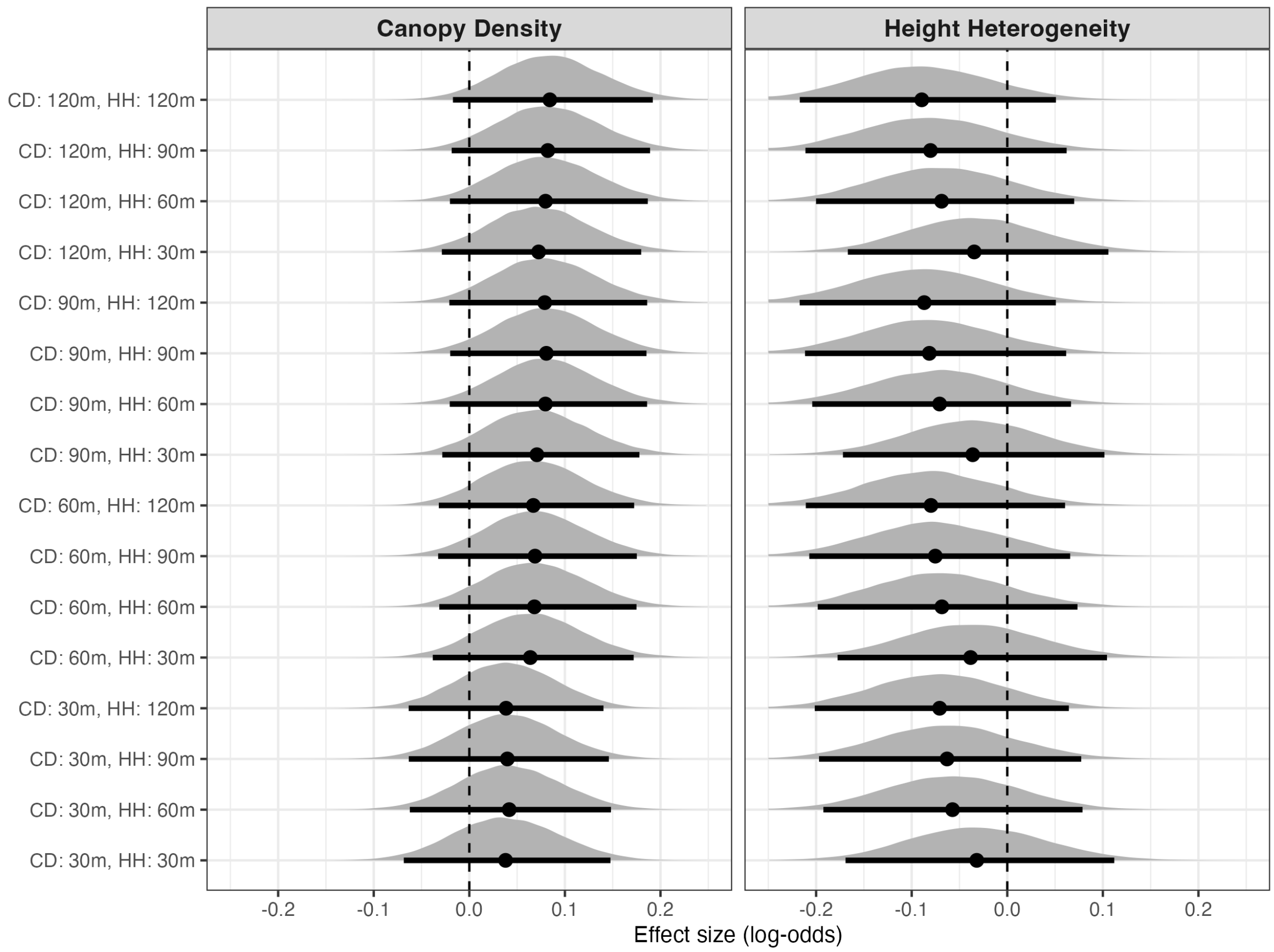

Supplementary Figure 11: Sensitivity check across 16 models varying the buffer radius used to compute canopy density and height heterogeneity. Each row is a model fit with a unique radius combination. Black markers represent the median of each distribution, bold and thin intervals surrounding the point estimate represent the 66% and 95% credible intervals, respectively. Source data are provided as a Source Data file.

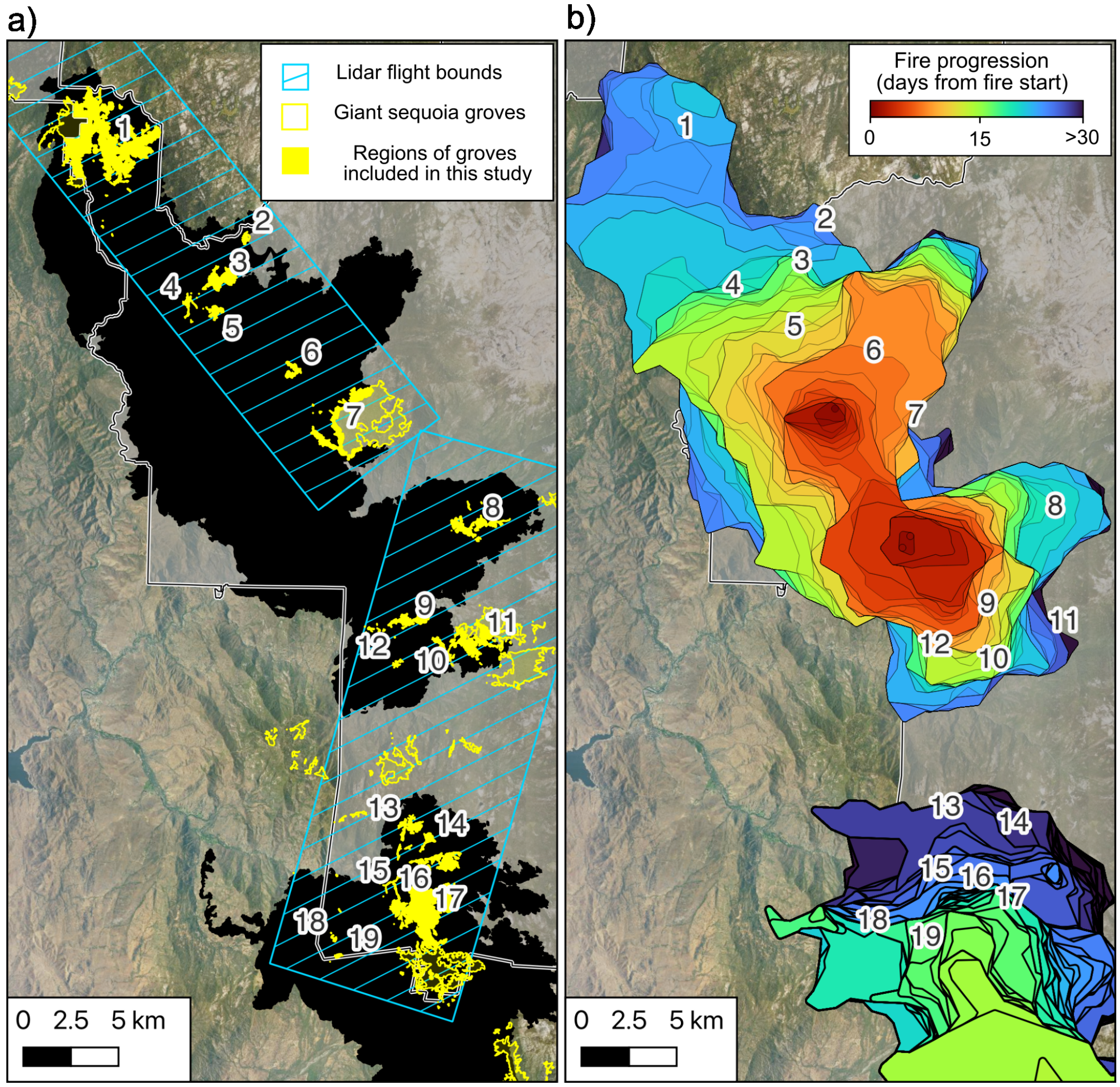

Supplementary Figure 12: Location of 19 groves in this study within Sequoia and Kings Canyon national parks (black line, white stroke) overlaid with airborne lidar surveys from 2015-2016 and the KNP + Castle Fire footprints. Also shown are VIIRS-derived daily fire perimeters from Chen et al. (2022). Grove numbers and names correspond to those used in Fig. 1 of the main text.

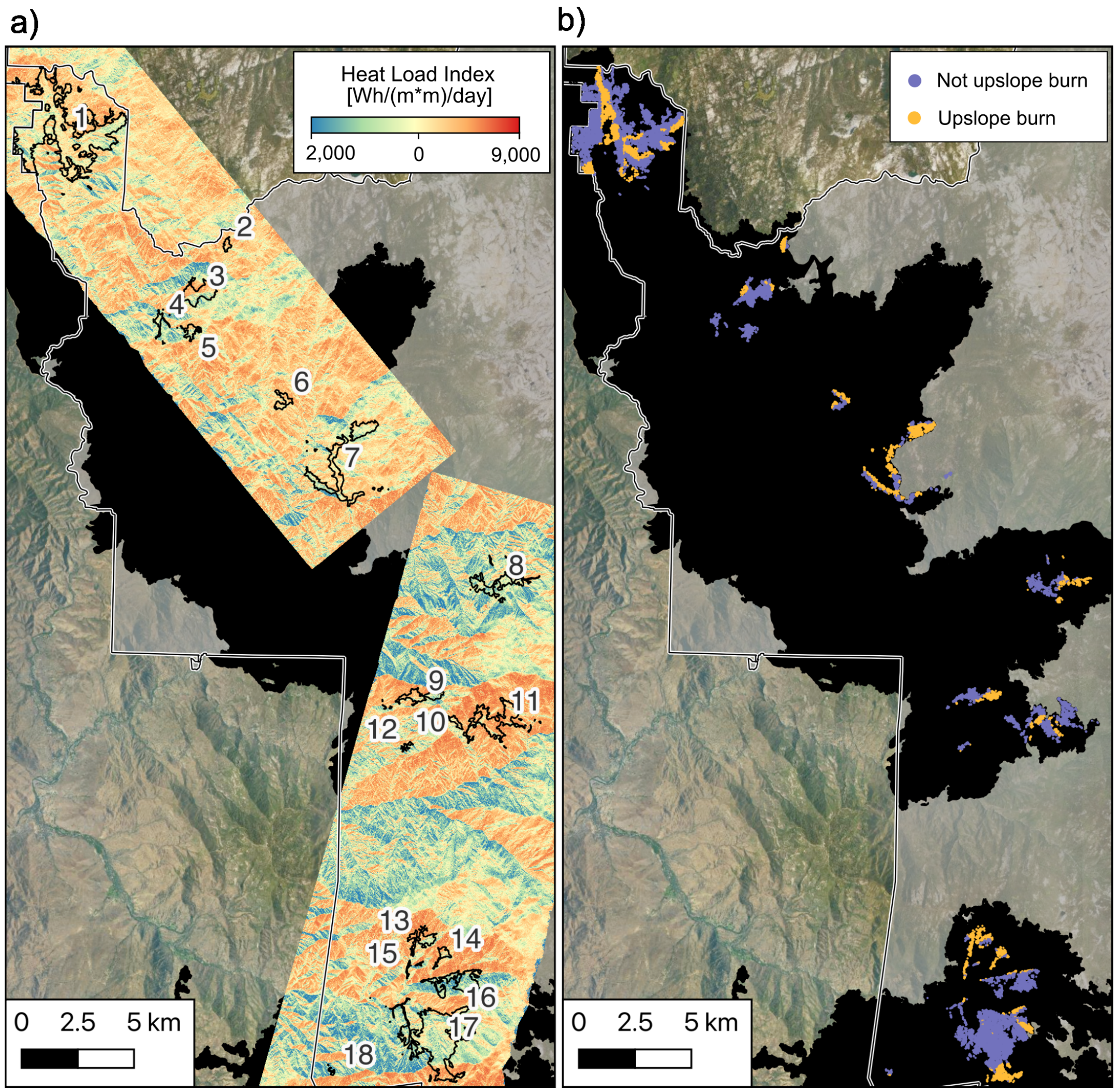

Supplementary Figure 13: Location of 19 groves in this study within Sequoia and Kings Canyon national parks (black line, white stroke) overlaid with lidar-derived Heat Load Index (HLI) and the tree-level upslope indicator (upslope = 1; orange marker).

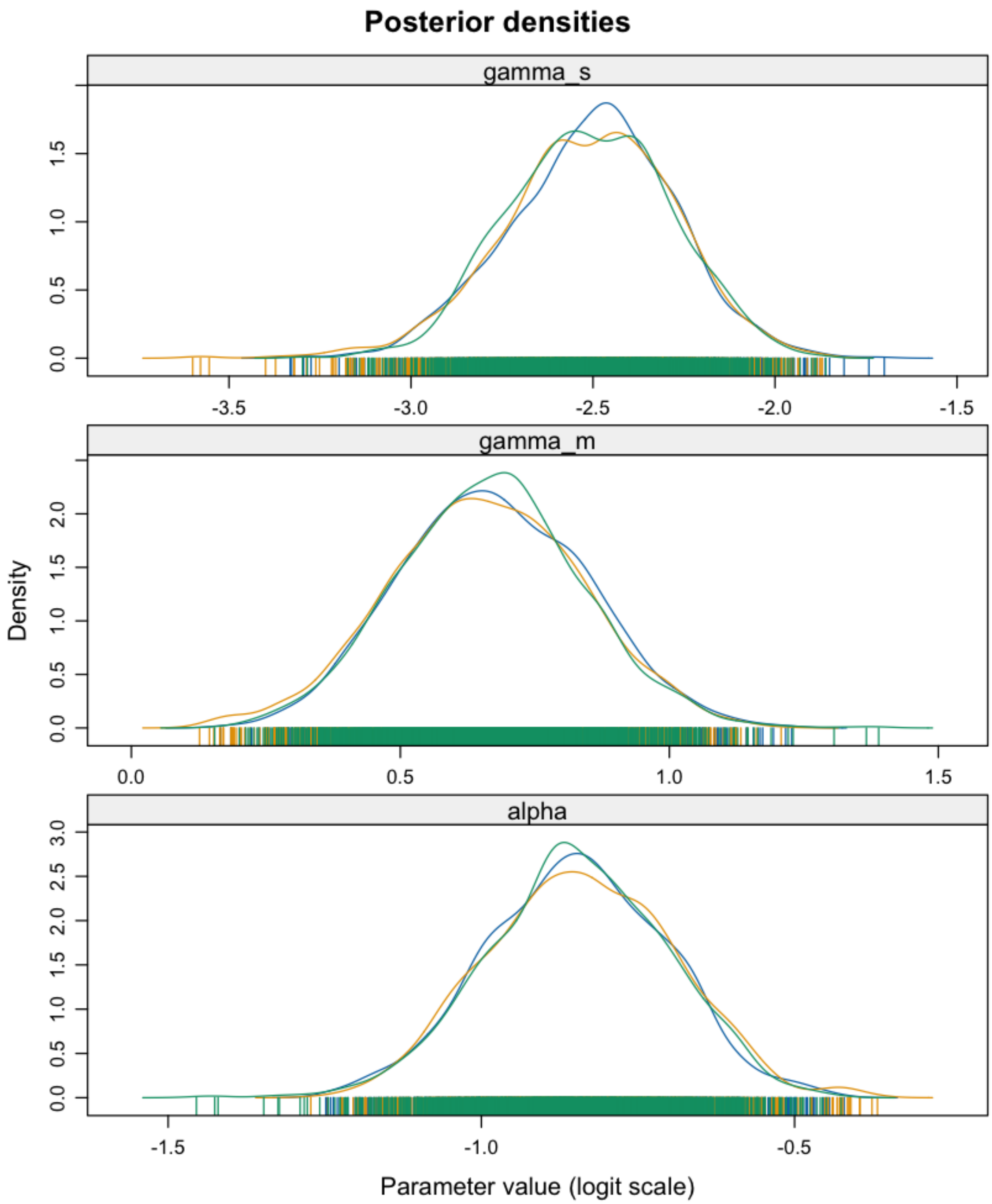

Supplementary Figure 14: Posterior densities for calibration model parameters α, γ_m, and γ_s showing distributions separated across three chains.

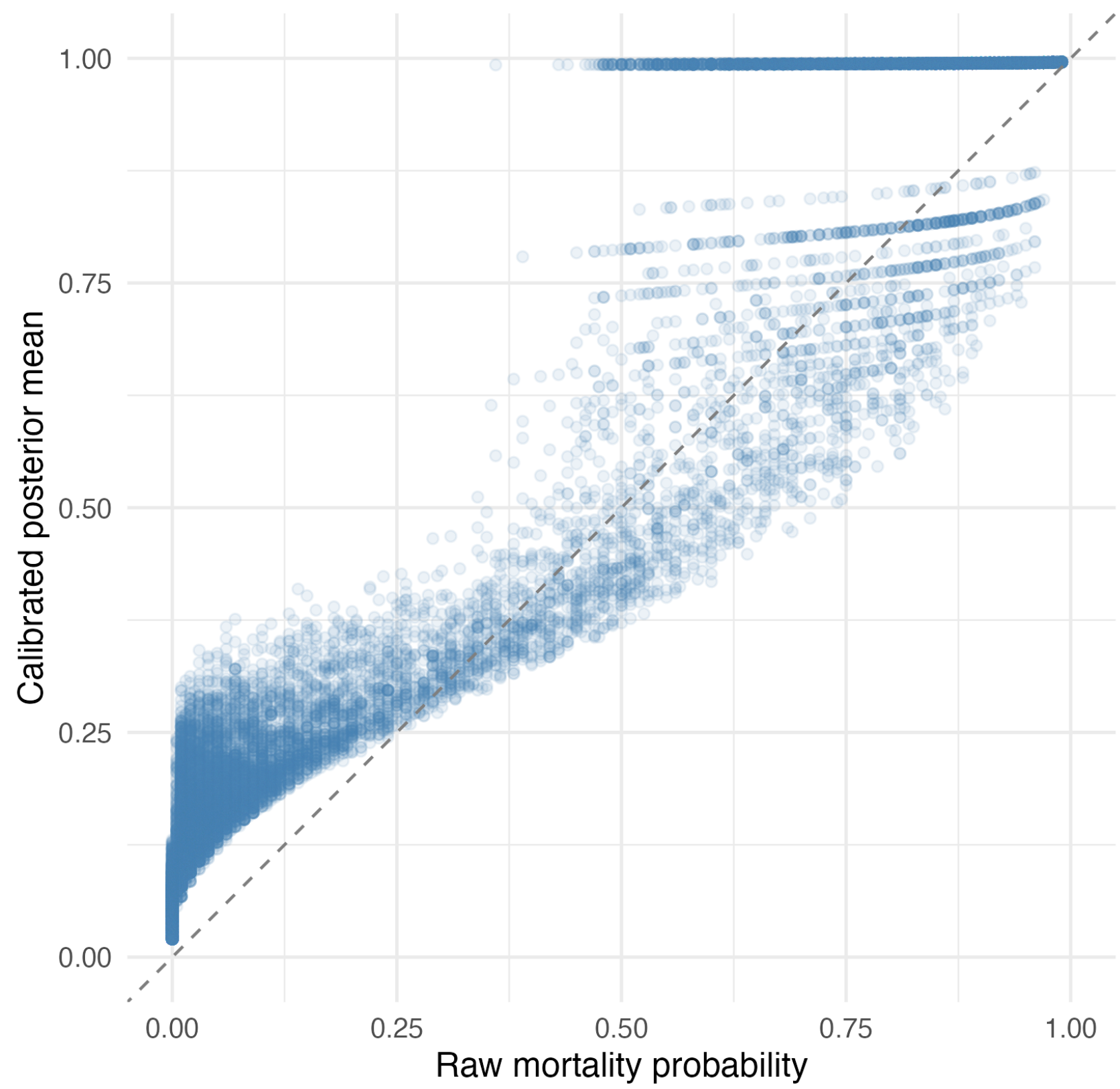

Supplementary Figure 15: Comparison of raw neural network mortality probabilities and calibrated posterior means for all giant sequoias. The dashed line represents 1:1 agreement.

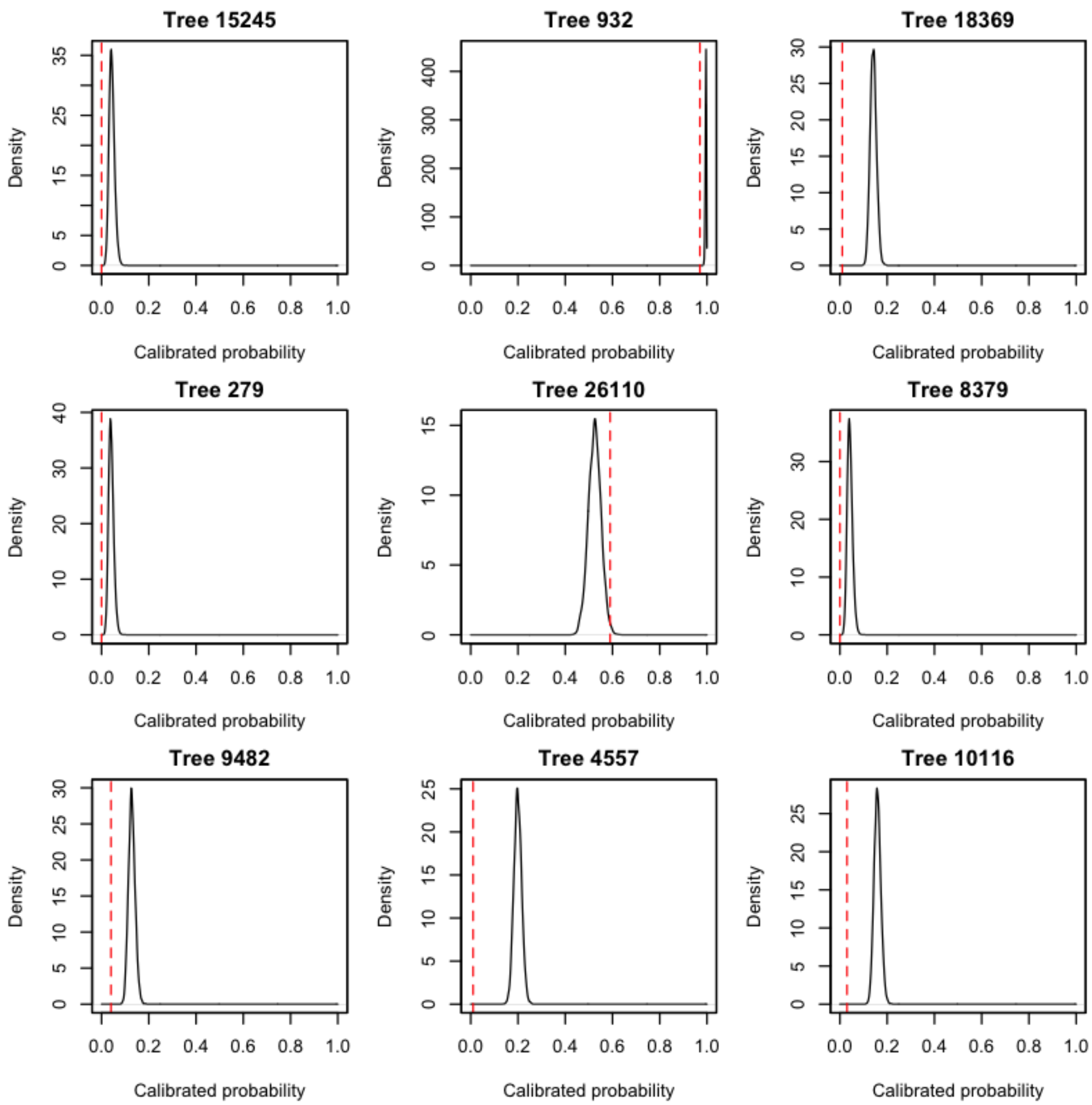

Supplementary Figure 16: Posterior distributions of calibrated mortality probabilities for nine random trees. Red dashed lines indicate raw (uncalibrated) neural network mortality probability point estimates for comparison.

Supplementary Tables

Supplementary Table 1: Model comparisons of goodness-of-fit across different spatial scales used to calculate forest structure metrics. LOOIC = Leave-one-out information criterion (lower values indicate better fit); elpd_loo = expected log pointwise predictive density; p_loo = effective number of parameters. All models show similar fit, indicating results are robust to choice of neighborhood radius.

Canopy Dens.	Height Het.	LOOIC (SE)	elpd_loo (SE)	p_loo (SE)
0–30 m	0–30 m	4857.3 (20.7)	-2428.7 (10.4)	918.4 (9.1)
0–30 m	0–60 m	4854.9 (20.7)	-2427.5 (10.4)	917.1 (9.1)
0–30 m	0–90 m	4855.9 (20.7)	-2428.0 (10.4)	917.7 (9.1)
0–30 m	0–120 m	4856.0 (20.8)	-2428.0 (10.4)	917.8 (9.2)
0–60 m	0–30 m	4857.3 (20.7)	-2428.6 (10.3)	918.9 (9.1)
0–60 m	0–60 m	4846.8 (21.3)	-2423.4 (10.7)	914.7 (9.2)
0–60 m	0–90 m	4858.0 (20.7)	-2429.0 (10.4)	919.8 (9.1)
0–60 m	0–120 m	4858.0 (20.8)	-2429.0 (10.4)	919.9 (9.2)
0–90 m	0–30 m	4857.0 (20.7)	-2428.5 (10.3)	919.0 (9.0)
0–90 m	0–60 m	4856.0 (20.7)	-2428.0 (10.3)	918.8 (9.1)
0–90 m	0–90 m	4856.0 (20.7)	-2428.0 (10.3)	919.0 (9.1)
0–90 m	0–120 m	4856.4 (20.8)	-2428.2 (10.4)	919.3 (9.1)
0–120 m	0–30 m	4856.5 (20.7)	-2428.3 (10.3)	919.0 (9.0)
0–120 m	0–60 m	4854.2 (20.7)	-2427.1 (10.3)	917.8 (9.0)
0–120 m	0–90 m	4856.0 (20.7)	-2428.0 (10.3)	919.0 (9.1)
0–120 m	0–120 m	4855.5 (20.7)	-2427.7 (10.4)	918.9 (9.1)
