# Supplementary material for: Previous prescribed burns saved thousands of ancient sequoias during historically unprecedented wildfires
Source: Nat Commun. 2026 Jul 11;17:8558. doi: 10.1038/s41467-026-75418-6 (PMC13483421; doi:10.1038/s41467-026-75418-6)
Supplement: Supplementary file 2 [file 41467_2026_75418_MOESM2_ESM.pdf]

nature portfolio

Corresponding author(s): Dan Dixon

Last updated by author(s): May 26, 2026

Reporting Summary

Nature Portfolio wishes to improve the reproducibility of the work that we publish. This form provides structure for consistency and transparency in reporting. For further information on Nature Portfolio policies, see our Editorial Policies and the Editorial Policy Checklist.

Statistics

For all statistical analyses, confirm that the following items are present in the figure legend, table legend, main text, or Methods section.

n/a	Confirmed	
☐	☒	The exact sample size (*n*) for each experimental group/condition, given as a discrete number and unit of measurement
☐	☒	A statement on whether measurements were taken from distinct samples or whether the same sample was measured repeatedly
☒	☐	The statistical test(s) used AND whether they are one- or two-sided<br>*Only common tests should be described solely by name; describe more complex techniques in the Methods section.*
☐	☒	A description of all covariates tested
☒	☐	A description of any assumptions or corrections, such as tests of normality and adjustment for multiple comparisons
☐	☒	A full description of the statistical parameters including central tendency (e.g. means) or other basic estimates (e.g. regression coefficient) AND variation (e.g. standard deviation) or associated estimates of uncertainty (e.g. confidence intervals)
☒	☐	For null hypothesis testing, the test statistic (e.g. *F*, *t*, *r*) with confidence intervals, effect sizes, degrees of freedom and *P* value noted<br>*Give P values as exact values whenever suitable.*
☐	☒	For Bayesian analysis, information on the choice of priors and Markov chain Monte Carlo settings
☐	☒	For hierarchical and complex designs, identification of the appropriate level for tests and full reporting of outcomes
☒	☐	Estimates of effect sizes (e.g. Cohen's *d*, Pearson's *r*), indicating how they were calculated

Our web collection on statistics for biologists contains articles on many of the points above.

Software and code

Policy information about availability of computer code

Data collection	PlanetScope satellite imagery was acquired via the Planet Labs API. Airborne lidar was collected by the Carnegie Airborne Observatory. Giant sequoia stem locations were obtained from the Sequoia Tree Inventory. Prescribed burn perimeters and fire progression data were obtained from existing spatial databases.
Data analysis	Tree-level mortality was classified using a previously published spatio-temporal convolutional neural network implemented in Python v3.11 using TensorFlow v2.11 (Dixon et al. 2023). Crown segmentation and lidar processing were performed using the lidR package in R. Bayesian statistical modeling was conducted in R using JAGS. Custom R scripts were used for calibration, ensemble model fitting, counterfactual simulations, and figure generation.

For manuscripts utilizing custom algorithms or software that are central to the research but not yet described in published literature, software must be made available to editors and reviewers. We strongly encourage code deposition in a community repository (e.g. GitHub). See the Nature Portfolio guidelines for submitting code & software for further information.

Data

Policy information about availability of data

All manuscripts must include a data availability statement. This statement should provide the following information, where applicable:

- Accession codes, unique identifiers, or web links for publicly available datasets
- A description of any restrictions on data availability
- For clinical datasets or third party data, please ensure that the statement adheres to our policy

Datasets required to reproduce the analyses presented in this study are publicly archived through Open Science Framework at https://doi.org/10.17605/OSF.IO/83S2U. Raw PlanetScope imagery is available under license from Planet Labs and is therefore not publicly redistributed. Source data are provided with this paper.

Code supporting the analyses, statistical modeling, scenario simulations, and figure generation presented in this study is publicly available through GitHub and archived through Zenodo at https://doi.org/10.5281/zenodo.20143683.

Research involving human participants, their data, or biological material

Policy information about studies with human participants or human data. See also policy information about sex, gender (identity/presentation), and sexual orientation and race, ethnicity and racism.

Reporting on sex and gender	*Use the terms sex (biological attribute) and gender (shaped by social and cultural circumstances) carefully in order to avoid confusing both terms. Indicate if findings apply to only one sex or gender; describe whether sex and gender were considered in study design; whether sex and/or gender was determined based on self-reporting or assigned and methods used.*<br>*Provide in the source data disaggregated sex and gender data, where this information has been collected, and if consent has been obtained for sharing of individual-level data; provide overall numbers in this Reporting Summary. Please state if this information has not been collected.*<br>*Report sex- and gender-based analyses where performed, justify reasons for lack of sex- and gender-based analysis.*
Reporting on race, ethnicity, or other socially relevant groupings	*Please specify the socially constructed or socially relevant categorization variable(s) used in your manuscript and explain why they were used. Please note that such variables should not be used as proxies for other socially constructed/relevant variables (for example, race or ethnicity should not be used as a proxy for socioeconomic status).*<br>*Provide clear definitions of the relevant terms used, how they were provided (by the participants/respondents, the researchers, or third parties), and the method(s) used to classify people into the different categories (e.g. self-report, census or administrative data, social media data, etc.)*<br>*Please provide details about how you controlled for confounding variables in your analyses.*
Population characteristics	*Describe the covariate-relevant population characteristics of the human research participants (e.g. age, genotypic information, past and current diagnosis and treatment categories). If you filled out the behavioural & social sciences study design questions and have nothing to add here, write "See above."*
Recruitment	*Describe how participants were recruited. Outline any potential self-selection bias or other biases that may be present and how these are likely to impact results.*
Ethics oversight	*Identify the organization(s) that approved the study protocol.*

Note that full information on the approval of the study protocol must also be provided in the manuscript.

Field-specific reporting

Please select the one below that is the best fit for your research. If you are not sure, read the appropriate sections before making your selection.

☐ Life sciences ☐ Behavioural & social sciences ☒ Ecological, evolutionary & environmental sciences

For a reference copy of the document with all sections, see nature.com/documents/nr-reporting-summary-flat.pdf

Ecological, evolutionary & environmental sciences study design

All studies must disclose on these points even when the disclosure is negative.

Study description	We estimated wildfire mortality outcomes for 26,403 giant sequoias across 19 groves following the 2020 Castle and 2021 KNP Complex wildfires in Sequoia and Kings Canyon national parks, California. Using a Bayesian statistical framework, we modeled the effects of prescribed burn history and lidar-derived forest structure on individual tree mortality, and simulated counterfactual mortality outcomes under alternative prescribed burn scenarios.
Research sample	All giant sequoias (Sequoiadendron giganteum) greater than 20 m in height within the Castle and KNP Complex wildfire perimeters in Sequoia and Kings Canyon national parks were included, totaling 26,403 individuals across 19 groves. Stem locations were obtained from the Sequoia Tree Inventory, a comprehensive spatial database of individual sequoias surveyed in the 1960s–1970s with

coordinates refined using airborne lidar and imaging surveys in 2015–2017. The sample represents the near-complete population of mature giant sequoias affected by these wildfires.

Sampling strategy	No sample size calculation was performed. All mapped sequoias meeting inclusion criteria within the fire perimeters were included. For statistical modeling, spatially balanced random subsets of 3,501 trees were constructed by randomly selecting one tree per 90×90 m grid cell to reduce spatial autocorrelation. This procedure was repeated independently for each of 500 model fits to capture sampling uncertainty.
Data collection	Giant sequoia stem locations were obtained from the existing Sequoia Tree Inventory database. Post-fire mortality and survival status for a subset of 1,566 trees was recorded by field observers in 2022–2023, who assessed proportional crown scorch and torch effects. Satellite-derived mortality probabilities were generated from 3 m PlanetScope imagery using a previously published deep learning classifier. Pre-fire forest structure was characterized from airborne lidar collected by the Carnegie Airborne Observatory in 2015–2016. Prescribed burn perimeters and fire progression data were obtained from existing spatial databases.
Timing and spatial scale	Field observations were collected in 2022–2023, one to two years following the 2020 Castle and 2021 KNP Complex wildfires. PlanetScope imagery was collected in pre-fire (2019 for Castle, 2020 for KNP) and post-fire years (2021 for Castle, 2022 for KNP). Airborne lidar was collected in August 2015 and July 2016. The study spans 19 sequoia groves within Sequoia and Kings Canyon national parks across elevations of 1,100–2,400 m in the southern Sierra Nevada, California.
Data exclusions	Trees observed as dead before the 2020–2021 wildfires were excluded. Trees within 30 m of wildfire perimeter edges were excluded to avoid edge effects. Trees with more than 90% of their PlanetScope pixels classified as shrub or non-woody were excluded as likely coordinate errors (663 trees). Multi-stem clusters were treated as single individuals. Only trees greater than 20 m in height were included. All exclusion criteria were pre-established prior to analysis.
Reproducibility	All analyses were conducted using documented code and will be made publicly available upon acceptance. Model convergence was verified using Rhat statistics (<1.01) and visual inspection of trace plots. Results were validated through five-fold cross-validation of the calibration model and sensitivity analyses evaluating robustness to alternative model specifications, treatment window definitions, and spatial neighborhood sizes.
Randomization	Trees were not experimentally allocated to treatment groups. Prescribed burn treatment reflects historical management decisions made by Sequoia and Kings Canyon national parks prior to and independent of this study. Grove-level random intercepts were included in all models to account for non-random spatial clustering of trees within groves.
Blinding	Blinding was not relevant to this study. Field observers recorded mortality status independently of the satellite-derived classifications. Satellite-derived mortality probabilities were generated from a previously published model applied uniformly across all trees.

Did the study involve field work? ☐ Yes ☒ No

Reporting for specific materials, systems and methods

We require information from authors about some types of materials, experimental systems and methods used in many studies. Here, indicate whether each material, system or method listed is relevant to your study. If you are not sure if a list item applies to your research, read the appropriate section before selecting a response.

Materials & experimental systems

n/a	Involved in the study
☒	☐ Antibodies
☒	☐ Eukaryotic cell lines
☒	☐ Palaeontology and archaeology
☒	☐ Animals and other organisms
☒	☐ Clinical data
☒	☐ Dual use research of concern
☒	☐ Plants

Methods

n/a	Involved in the study
☒	☐ ChIP-seq
☒	☐ Flow cytometry
☒	☐ MRI-based neuroimaging

Plants

Seed stocks	*Report on the source of all seed stocks or other plant material used. If applicable, state the seed stock centre and catalogue number. If plant specimens were collected from the field, describe the collection location, date and sampling procedures.*
Novel plant genotypes	*Describe the methods by which all novel plant genotypes were produced. This includes those generated by transgenic approaches, gene editing, chemical/radiation-based mutagenesis and hybridization. For transgenic lines, describe the transformation method, the number of independent lines analyzed and the generation upon which experiments were performed. For gene-edited lines, describe the editor used, the endogenous sequence targeted for editing, the targeting guide RNA sequence (if applicable) and how the editor was applied.*
Authentication	*Describe any authentication procedures for each seed stock used or novel genotype generated. Describe any experiments used to assess the effect of a mutation and, where applicable, how potential secondary effects (e.g. second site T-DNA insertions, mosiacism, off-target gene editing) were examined.*
