# Supplementary material for: Previous prescribed burns saved thousands of ancient sequoias during historically unprecedented wildfires
Source: Nat Commun. 2026 Jul 11;17:8558. doi: 10.1038/s41467-026-75418-6 (PMC13483421; doi:10.1038/s41467-026-75418-6)
Supplement: Supplementary file 3 [file 41467_2026_75418_MOESM3_ESM.pdf]

nature portfolio

Peer Review File

Previous prescribed burns saved thousands of ancient sequoias during historically unprecedented wildfires

Corresponding Author: Dr Dan Dixon

This file contains all reviewer reports in order by version, followed by all author rebuttals in order by version.

Version 0:

Reviewer comments:

Reviewer #1

(Remarks to the Author)
The manuscript examines mortality of the iconic giant sequoia in the aftermath of extreme fires and models the contribution of prescribed burning to tree survival taking into account a number of other variables, e.g. that reflect fire behaviour. The work is admirable, first of all considering the the variety and sophisticated combination of the methods employed, including novel (as far as I can tell) applications of remote sensing. A few minor comments/issues follow.

L75-81. Nice description of the limitations of fire severity assessment based on spectral change.

Fig. 2. Typo “LIDAR-dervied”.

L137. Ladder fuels have already been defined earlier in the ms.

L214-230. Here we learn about some results not presented in the results section …

L233. Dense canopies promote horizontal fire spread in the canopy, rather than vertical spread.

L255. I suspect that this lack of influence of forest structure could also be an outcome of the surface fuel complex structure. I suppose fire behaviour in these forests is dominated by compact litter and downed dead woody fuels, i.e. relatively modest flame sizes and fire intensity that secondarize the role of vertical fuel continuity: the amount of discontinuity does not need to be much to hinder crown fire. Please address.

L355. VIIRS resolution is 250 m and fire perimeters are delineated at 24-hour intervals. Isn’t this too coarse in space and time to relate to whether or not a particular tree was subjected to an upslope fire run?

L387. So, the authors did not wait for the trees to actually die and defined a mortality threshold based on crown injury. Why >90%? Trees of fire-resistant species can often survive higher levels of scorch and crown scorch does not necessarily equate to crown kill, as buds can survive and produce foliage in the following spring.

L133. Not totally sure about what is meant by “spatially thinned”.

Reviewer #2

(Remarks to the Author)

First, I will acknowledge that the Bayesian statistics are outside of my area of expertise, and I cannot evaluate the application of this approach. However, I find the remote sensing and general modeling components to be sound, and very much appreciate the sensitivity analyses and reporting of confidence intervals throughout that bring credence to the results.

This is an extremely thorough and well-written paper, on top of being much needed. Thank you to the authors for submitting a manuscript that was polished and easy to follow, with seemingly every base covered in terms of potential explanations for the observed mortality, and clear boundaries on interpretation of results. The figures and tables were excellent in supporting

the narrative.

Critically, these findings address fundamental questions currently confronting forest management, including: can the Giant Sequoia be saved despite climate change? Does prescribed fire or forest thinning have the greater capacity to protect these ancient trees? This paper utilizes novel data sets to look at the survival of tens of thousands of individual giant sequoias in recent extreme wildfires and tests every potential contributor to mortality to answer these questions. The analysis is quite thorough, and the results leave no doubt as to the utility of prescribed fire to reduce sequoia mortality. This is important because, as the manuscript notes, there is substantial anecdotal evidence on the utility of prescribed fire, but it is critical to quantify its effects and address other potential contributing factors.

I have a few monitor comments and suggestions:

Minor point – Isn't Sequoia and Kings Canyon National Parks capitalized in entirety (because it is the proper name)?

The paragraph from lines 73-86 (about RdNBR) feels out of place and excessive in an otherwise elegant and streamlined paper, as if the authors are trying to pre-emptively placate the burn severity crowd, and I would suggest it is not needed. This could easily be replaced by a very simple sentence stating that 30m resolution remote sensing commonly used to assess landscape fire effects is inadequate to resolve individual tree-scale mortality and would preface the subsequent paragraph introducing the use of 3m PlanetScope data.

Line 96: "photosynthetic area" … is this equivalent to "leaf area"? I'm more familiar with the latter term, and was unclear if there was a difference.

Page 10, Figure 4 caption: missing a closing parentheses at the end of the caption.

Reviewer #3

(Remarks to the Author)
The study applies a number of sophisticated methods to quantify the impacts of prior fire management strategies to tree-level mortality outcomes in giant sequoia trees during two recent, extraordinary fire events. The approach leverages a machine-learning model that was recalibrated for this species, individual tree surveys post-fire, airborne lidar data, grove stem maps, and a few other data sources. The main analysis involves post-fitting counterfactual simulations of mortality outcomes. These results suggest previous prescribed burning treatments were very effective at reducing ultimate mortality of trees in treated areas, and that this effect is stronger than any other variables we expect may play a role.

I think the study is quite important for a number of reasons, the most obvious being that it clearly demonstrates the conservation value of prescribed burns, which have sometimes been controversial in mainstream media and public forums. That this study does it for an extremely well recognized and critically important tree species makes it more valuable. The rigorous statistical analysis an extensive remotely sensed and ground truthed datasets are also a strength. I have been citing various webpages as evidence of giant sequoia mortality rates in the KNP and Castle fires (such as the reference #15 in the main text). It will be nice to have this paper to cite in the future.

High level comments.
1) The utility of the counterfactuals depends on the fit of the model from which they were generated. Brier score and F1 score are reported in the supplement. While these are good, they are a bit hard to interpret in this context, given multiple sites and groves, and they are not reported in the main text. I think the main text would benefit from some clear description of model fit. I am not sure if an R2 is appropriate or possible to calculate, though I know that more traditional tools such as random forests report their own R2. Such a metric would help contextualize the process uncertainty of the counterfactuals, in addition to the existing uncertainty propagation scheme.
2) Some more description of the machine-learning model would be useful, either in the main text or the supplement. It is simply referenced, without explanation to required software packages or implementation details. This weakens one of the discussions' claims that the approach is "broadly transferable".

Minor comments.
L14. Opening seems somewhat exaggerated for a scientific article. "worlds most fire adapted" seems to be a difficult claim to evaluate.
L24. A bit unclear, missing comma or other symbol
L31. Just a note that the control treatment / category is not clear from the abstract
L37. Extensive mortality of … associated biodiversity." Perhaps rephrase.
L48. Replace 'defenses' with 'adaptations'
L58-60. And also occasionally hostile public opinion!

L327. Any bias in the locations of this validation dataset or were these distributed across most of the SEKI?
L342. Unclear what is meant by "user" and "producer"
L400. Please state types of uncertainty here or below. i.e. location uncertainty, mortality uncertainty, identity uncertainty?
L416. I believe this is a the first mention of the burn treatment indicator variable. Could the authors please add some text earlier that explains how burn treatment is represented in the model approach?
L426. Fig. S9.

L435. Some typo here, repeated 90m
L513. So this is “prescribed_burn” in the model? Can this be stated?

L107. What are the software packages used for the CNN?
L148. Please report 95% CI to be consistent with rest of paragraph. Reporting of a single 80% CI for this effect alone is confusing and potentially misleading without additional justification for this choice. Also, in this paragraph, only the prescribe burning effect is translated into a % change in mortality probability, while the others are only presented as log-odds in the figure. Reporting of the raw scale % would be useful for the other effects to compare (e.g. upslope burn).
-Also, there is a mix of presentation of credible intervals and confidence intervals here. I might suggest to refer to all as CI, as hardly anyone is interested in the distinction and others find CrI confusing.
L269. “The approach is broadly transferable.” I think some more nuanced discussion would be necessary to make this claim. I might suggest that one of the reasons that approach is viable for giant sequoia forests is that canopies are large and forests are relatively sparse, compared to say, a mixed conifer forest where canopies are <3 m wide and tree density is much higher. I believe the approach may face challenges with resolution in other forests. There were some issues with coordinates not matching tree locations precisely (Quality 2/3) even here. Furthermore, a major strength of the current study is the independent tree-level validation.

Version 1:

Reviewer comments:

Reviewer #1

(Remarks to the Author)
The authors adequately addressed the reviewers' concerns and I think it is ready for acceptance. This is a nice and timely contribution.

Reviewer #2

(Remarks to the Author)
My comments were addressed satisfactorily.

Reviewer #3

(Remarks to the Author)
I thank the authors for their thorough responses to each of my comments. I appreciate the clarifications of some methodologies which I had not quite understood during my first review.

Open Access This Peer Review File is licensed under a Creative Commons Attribution 4.0 International License, which permits use, sharing, adaptation, distribution and reproduction in any medium or format, as long as you give appropriate credit to the original author(s) and the source, provide a link to the Creative Commons license, and indicate if changes were made.

In cases where reviewers are anonymous, credit should be given to 'Anonymous Referee' and the source.
The images or other third party material in this Peer Review File are included in the article’s Creative Commons license, unless indicated otherwise in a credit line to the material. If material is not included in the article’s Creative Commons license and your intended use is not permitted by statutory regulation or exceeds the permitted use, you will need to obtain permission directly from the copyright holder.
To view a copy of this license, visit https://creativecommons.org/licenses/by/4.0/

Reviewer #1

The manuscript examines mortality of the iconic giant sequoia in the aftermath of extreme fires and models the contribution of prescribed burning to tree survival taking into account a number of other variables, e.g. that reflect fire behaviour. The work is admirable, first of all considering the the variety and sophisticated combination of the methods employed, including novel (as far as I can tell) applications of remote sensing. A few minor comments/issues follow.

- ➢ We thank the reviewer for their assessment of the manuscript. We appreciate the thoughtful comments and have addressed each below.

L75-81. Nice description of the limitations of fire severity assessment based on spectral change.

- ➢ Thank you.

Fig. 2. Typo “LIDAR-dervied”.

- ➢ Thanks for catching this. Throughout the revised manuscript, we consistently use lowercase to refer to "lidar".

L137. Ladder fuels have already been defined earlier in the ms.

- ➢ That's a good point. In the revised version, we now clearly define ladder fuels as "vertically continuous live or dead vegetation facilitating upward fire movement into tree crowns" in the Introduction on L72 and removed the L137 definition from the Results section.

L214-230. Here we learn about some results not presented in the results section …

- ➢ We thank the reviewer for this comment. In the Discussion, we refer to findings (sensitivity analyses) that are presented in the Results section (L224–225; Supplementary Figs. 11–16). However, to avoid confusion and redundancy, we have revised the Discussion text to emphasize synthesis and interpretation of these results rather than introducing new findings. However, we want to remind the reader of those results to discuss them further. The new text reads:
  - ➢ L223-225: The prescribed burn effect remained stable across multiple sensitivity checks: expanding the 224 recent treatment window from ≤10 to ≤15 years, removing structural covariates, or altering model 225 structure all yielded nearly identical reductions in mortality odds (Supplementary Figs. 6, 11).

L233. Dense canopies promote horizontal fire spread in the canopy, rather than vertical spread.

- ➢ We thank the reviewer for this clarification. We agree that dense canopies primarily facilitate fire spread within the canopy, whereas vertical spread from the surface into the canopy is more directly associated with ladder fuels. We have revised the text

accordingly to distinguish these mechanisms. The revised sentence now reads on L241-243: “Ecological theory and prior studies indicate that elevated ladder fuels promote vertical flame spread into tree crowns, while dense canopies facilitate fire spread within the canopy, and more heterogeneous stands with canopy gaps tend to resist severe fire.”

L255. I suspect that this lack of influence of forest structure could also be an outcome of the surface fuel complex structure. I suppose fire behaviour in these forests is dominated by compact litter and downed dead woody fuels, i.e. relatively modest flame sizes and fire intensity that secondarize the role of vertical fuel continuity: the amount of discontinuity does not need to be much to hinder crown fire. Please address.

- ➢ We thank the reviewer for this insightful comment. We agree that fire behavior in giant sequoia groves is often strongly influenced by surface fuel complexes like compact litter and downed woody fuels. And that under such conditions relatively modest vertical fuel discontinuity may be sufficient to limit crown fire. In this context, variation in canopy structure or ladder fuels may exert less control over tree-level mortality than surface fuel conditions.
- ➢ In the revised Discussion, we clarify that although canopy structure and ladder fuels are expected to influence fire behavior in general, the weak effects of lidar-derived structure metrics in our analysis likely reflect limited additional explanatory power once prescribed fire and its associated reduction of surface and near-surface fuels is accounted for. We further note that under extreme fire weather, surface fuel loads may dominate fire behavior and mortality outcomes, reducing the ability of canopy-scale structural metrics to affect survival at the individual-tree scale. We emphasize that this interpretation is offered as a plausible explanation rather than a directly tested mechanism.
    - ➢ The revised text now reads on L213-217: “Our findings are consistent with surface fuels being a key driver of sequoia mortality risk: prescribed burning had a strong negative effect on mortality, while forest structure metrics showed little explanatory power (Fig. 3). This pattern indicates that the dominant effects of prescribed fire operate through surface and near-surface fuel attributes that are not fully captured by canopy-scale lidar metrics.”
    - ➢ Also on L243-248: “We therefore expected lidar-derived structure metrics to help explain variation in giant sequoia mortality. However, these metrics consistently offered minimal explanatory power, a pattern that held across multiple sensitivity checks. Under extreme fire weather, variation in surface fuel loads may exert a dominant influence on fire behavior and subsequent mortality, whereas local variability in canopy structure provides limited additional discriminatory power at the individual-tree scale.”

L355. VIIRS resolution is 250 m and fire perimeters are delineated at 24-hour intervals. Isn’t this too coarse in space and time to relate to whether or not a particular tree was subjected to an

upslope fire run?

- ➢ We thank the reviewer for raising this concern. We agree that VIIRS active fire detections are too coarse to resolve continuous fine-scale fire behavior at the level of individual trees. Accordingly, our analysis does not attempt to infer fire intensity, flame length, or precise timing of fire arrival at individual stems.
- ➢ Instead, we used an object-based fire progression dataset derived from VIIRS I-band detections that delineates the advancing fire front at 24-hour intervals. These fire progressions were used only to infer the dominant direction of fire spread (upslope or not) relative to local terrain. Given the spatial coherence of slope and fire runs at this scale, we treated upslope exposure as a binary indicator, avoiding finer-grained or continuous representations that would not be supported by the data.
- ➢ We acknowledge that this approach cannot capture small-scale variability in fire behavior, but we consider it a conservative and appropriate representation of fire spread direction given available observations. We have clarified this rationale and limitation in the Methods (L466):
    - ➢ "Given uncertainty in fire front position and timing at fine scales, upslope exposure was treated as a binary indicator rather than a continuous measure."

L387. So, the authors did not wait for the trees to actually die and defined a mortality threshold based on crown injury. Why >90%? Trees of fire-resistant species can often survive higher levels of scorch and crown scorch does not necessarily equate to crown kill, as buds can survive and produce foliage in the following spring.

- ➢ We thank the reviewer for raising this point. We agree that crown scorch alone does not necessarily imply mortality in fire-resistant species, including giant sequoia, particularly at moderate levels of injury. In this study, mortality was defined as >90% loss of green crown one year post-fire, which we use as a conservative proxy for mortality.
- ➢ From an aerial perspective, a >90% loss of green crown corresponds to near-complete loss of photosynthetically active foliage at the tree level, as small surviving foliage patches are difficult to reliably detect. As a result, this threshold is conservative and more likely to underestimate, rather than overestimate, true mortality. In ground-based assessments, comparable classifications would correspond to >95–99% foliage loss.
- ➢ This interpretation is supported by field studies showing that giant sequoias with very high levels of crown injury have a high probability of eventual mortality, with deaths continuing after the first post-fire year. For example, Shive et al. (2022) documented substantial delayed mortality among large sequoias with severe crown damage following wildfire. Consistent with these findings, one-year post-fire assessments tend to underestimate, rather than overestimate, eventual mortality.
- ➢ Our objective was to provide consistent, landscape-scale mortality estimates while

prioritizing specificity and acknowledging that some delayed mortality may not yet be expressed when observed by the satellite.

Shive, K. L. et al. (2022). Ancient trees and modern wildfires: Declining resilience to wildfire in the highly fire-adapted giant sequoia. Forest Ecology and Management 511.

L133. Not totally sure about what is meant by “spatially thinned”.

- ➢ We thank the reviewer for noting this ambiguity. By “spatially thinned,” we mean that we sampled spatially balanced random subsets of sequoias to reduce fine-scale spatial autocorrelation. We have revised the manuscript to replace this term at first use and to clarify the grid-based procedure used to construct these subsets in Results and Methods. These changes improve clarity without altering the analysis. Please see the following changes:
    - ➢ L136: "...fit to spatially balanced random subsets of sequoias (3,501 trees) to 137 reduce fine-scale spatial autocorrelation."
    - ➢ L414: "Each GLMM was fit to a spatially balanced subset 415 of trees (n = 3,501) and included a grove-level random intercept."
    - ➢ and L477: "To reduce spatial autocorrelation, we constructed spatially balanced random subsets of sequoias by overlaying a 90 × 90 m grid and randomly selecting one tree per cell (n = 3,501, or 13.2% of all trees), with the selection independently re-randomized for each model fit."

Reviewer 2:

First, I will acknowledge that the Bayesian statistics are outside of my area of expertise, and I cannot evaluate the application of this approach. However, I find the remote sensing and general modeling components to be sound, and very much appreciate the sensitivity analyses and reporting of confidence intervals throughout that bring credence to the results.

This is an extremely thorough and well-written paper, on top of being much needed. Thank you to the authors for submitting a manuscript that was polished and easy to follow, with seemingly every base covered in terms of potential explanations for the observed mortality, and clear boundaries on interpretation of results. The figures and tables were excellent in supporting the narrative.

Critically, these findings address fundamental questions currently confronting forest management, including: can the Giant Sequoia be saved despite climate change? Does prescribed fire or forest thinning have the greater capacity to protect these ancient trees? This paper utilizes novel data sets to look at the survival of tens of thousands of individual giant sequoias in recent extreme wildfires and tests every potential contributor to mortality to answer these questions. The analysis is quite thorough, and the results leave no doubt as to the utility of prescribed fire to reduce sequoia mortality. This is important because, as the manuscript notes, there is substantial anecdotal evidence on the utility of prescribed fire, but it is critical to

quantify its effects and address other potential contributing factors.

- ➢ Thank you for highlighting the broader relevance of these findings for forest management. We appreciate the reviewer's recognition of the value of quantifying prescribed fire effects on individual sequoia survival, particularly given the long reliance on anecdotal evidence.

Minor point – Isn't Sequoia and Kings Canyon National Parks capitalized in entirety (because it is the proper name)?

- ➢ Good question. We've decided to use "Sequoia and Kings Canyon national parks" because they are two separate parks and, in that case, there is no need to capitalize the entire name. We follow the convention used by researchers working within the parks (e.g., https://link.springer.com/article/10.1186/s42408-024-00316-5

The paragraph from lines 73-86 (about RdNBR) feels out of place and excessive in an otherwise elegant and streamlined paper, as if the authors are trying to pre-emptively placate the burn severity crowd, and I would suggest it is not needed. This could easily be replaced by a very simple sentence stating that 30m resolution remote sensing commonly used to assess landscape fire effects is inadequate to resolve individual tree-scale mortality and would preface the subsequent paragraph introducing the use of 3m PlanetScope data.

- ➢ That's a great point and thanks for raising it. We agree that a detailed discussion or critique of RdNBR is not necessary in this context. The key limitation of many existing approaches is their coarse spatial resolution, not the spectral indices themselves. Accordingly, we have revised the Introduction to remove specific discussion of RdNBR and instead clarify that commonly used ~30 m satellite-based fire severity metrics aggregate outcomes across mixed pixels and cannot resolve species-specific mortality at the scale of individual trees. Please see the new text on L80-85:
    - ➢ "However, these metrics aggregate outcomes across mixed pixels that contain multiple species and vegetation types, rather than resolving mortality of individual trees... severity classes correspond to wide and overlapping mortality ranges, limiting inference about treatment effectiveness for long-lived, fire-resistant species such as giant sequoia. Tree-level mortality datasets at finer spatial resolution are therefore needed to evaluate species-specific outcomes under extreme wildfire conditions."

Line 96: "photosynthetic area" … is this equivalent to "leaf area"? I'm more familiar with the latter term, and was unclear if there was a difference.

- ➢ We agree that the term "photosynthetic area" could be unclear. Our intent was to distinguish photosynthetically active crown material from scorched foliage that may remain present but is no longer functional. In the revised manuscript, we now use the term "photosynthetic crown foliage" to explicitly refer to green, photosynthetically active material in the crown, consistent with how mortality is inferred from spectral change one

year post-fire. Please find the new text on L96:

- ➢ "...and PlanetScope imagery to classify trees as live or dead based on >90% loss of photosynthetic crown foliage as observed in aerial imagery one year post-fire."

Page 10, Figure 4 caption: missing a closing parentheses at the end of the caption.

- ➢ Fixed

Reviewer #3

The study applies a number of sophisticated methods to quantify the impacts of prior fire management strategies to tree-level mortality outcomes in giant sequoia trees during two recent, extraordinary fire events. The approach leverages a machine-learning model that was recalibrated for this species, individual tree surveys post-fire, airborne lidar data, grove stem maps, and a few other data sources. The main analysis involves post-fitting counterfactual simulations of mortality outcomes. These results suggest previous prescribed burning treatments were very effective at reducing ultimate mortality of trees in treated areas, and that this effect is stronger than any other variables we expect may play a role.

I think the study is quite important for a number of reasons, the most obvious being that it clearly demonstrates the conservation value of prescribed burns, which have sometimes been controversial in mainstream media and public forums. That this study does it for an extremely well recognized and critically important tree species makes it more valuable. The rigorous statistical analysis an extensive remotely sensed and ground truthed datasets are also a strength. I have been citing various webpages as evidence of giant sequoia mortality rates in the KNP and Castle fires (such as the reference #15 in the main text). It will be nice to have this paper to cite in the future.

1) The utility of the counterfactuals depends on the fit of the model from which they were generated. Brier score and F1 score are reported in the supplement. While these are good, they are a bit hard to interpret in this context, given multiple sites and groves, and they are not reported in the main text. I think the main text would benefit from some clear description of model fit. I am not sure if an R2 is appropriate or possible to calculate, though I know that more traditional tools such as random forests report their own R2. Such a metric would help contextualize the process uncertainty of the counterfactuals, in addition to the existing uncertainty propagation scheme.

- ➢ Thank you for this comment. We agree that clearer discussion of model adequacy is important for interpreting the counterfactual analyses, and we have made targeted revisions to address this concern.
- ➢ First, we clarified the role of the performance metrics reported in the Supplementary Information. We now explicitly state that the Brier score and F1 score evaluate the satellite-based calibration model, not the downstream GLMM ensemble used for inference and counterfactual simulations. In the main Results, we revised the calibration paragraph to read (L122–124):

- ➢ "Across five-fold cross-validation on the field dataset, the calibration phase yielded a mean F1 score of 0.844, compared to 0.813 for the uncalibrated model (Supplementary Methods)."
- ➢ In the Supplementary Methods, we added a corresponding clarification noting that these metrics assess calibration performance only and do not evaluate the GLMM ensemble.

➢ Second, to directly address the adequacy of the GLMM ensemble underlying the counterfactual simulations, we evaluated its discriminatory performance using the area under the receiver operating characteristic curve (AUC), calculated against independent, ground-validated sequoia mortality observations. For each of the 500 ensemble draws, AUC was computed using the subset of trees with field observations available within that spatially balanced sample (median = 233 trees per draw). We did not report R^2 because variance-based measures are less informative for hierarchical Bernoulli models, whereas AUC provides a direct and interpretable measure of discrimination between observed mortality and survival outcomes. Across the ensemble, the GLMM showed good discriminatory performance, with a median AUC of 0.82 (95% interval: 0.72–0.90). We report this result in the main Results (L146–148):

- ➢ "When evaluated against the independent field-validated sequoia mortality observations available within each subset (median = 233 trees), the GLMM ensemble showed good discrimination, with a median AUC of 0.82 across 500 ensemble draws (95% CI: 0.72–0.90; Supplementary Methods)."
- ➢ We also added a brief subsection ("GLMM ensemble discriminatory performance") to the Supplementary Methods describing this evaluation. That text reads: "All performance metrics reported in this subsection (F1 score, Brier score, recall, and precision) evaluate the calibration model applied to the independent field dataset and do not assess the downstream GLMM ensemble."

➢ Finally, we revised language throughout the manuscript to avoid over-interpreting the GLMM ensemble as a predictive model. References to "prediction" were replaced with "simulation" where appropriate, and counterfactual results are now consistently described as simulated outcomes conditional on the fitted model and observed data structure.

- ➢ For example, on L159 we write: "Using posterior draws from the GLMM ensemble, we simulated giant sequoia mortality under two 160 counterfactual management scenarios, conditional on observed fire weather and landscape context."

➢ Together, these revisions clarify the role of each modeling component, provide a transparent assessment of GLMM adequacy relevant to the counterfactual analyses, and align the presentation with the simulation-based causal framework used in the

study.

2) Some more description of the machine-learning model would be useful, either in the main text or the supplement. It is simply referenced, without explanation to required software packages or implementation details. This weakens one of the discussions' claims that the approach is "broadly transferable".

- ➢ Thanks for raising this point. In the new version, we include a full paragraph in the Supplementary describing the tree mortality detection framework. It's included here:
    - ➢ "The satellite mortality model used in this study is a previously published spatio-temporal convolutional neural network (ST-CNN) designed to detect canopy-scale tree and shrub mortality from PlanetScope imagery at 3-m resolution (Dixon2023SatelliteLearning). The model jointly exploits spatial context and pre- and post-disturbance temporal dynamics using monthly PlanetScope composites, allowing it to distinguish heterogeneous patterns of survival and mortality at the scale of individual canopies. Training labels were generated using a semi-automated workflow that combines pre-fire airborne lidar crown segmentation with post-fire interpretation of very high-resolution aerial imagery, producing over one million labeled tree and shrub canopies across multiple California wildfires. The network was implemented in Python using TensorFlow/Keras and outputs class probabilities for tree mortality, tree survival, shrub mortality, shrub survival, and non-woody cover. In this study, we leverage the published model outputs and calibrate them specifically for giant sequoias to improve probabilistic accuracy and enable full uncertainty propagation through subsequent statistical analyses."

L14. Opening seems somewhat exaggerated for a scientific article. "worlds most fire adapted" seems to be a difficult claim to evaluate.

- ➢ We agree that this wording was overly strong. We have revised the text to be more conservative. The abstract now reads (L15): "...underscoring the vulnerability of even highly fire-adapted trees to ongoing environmental changes."

L24. A bit unclear, missing comma or other symbol

- ➢ Thanks for this comment. We've cleaned up the text to separate the estimated mortality count from the percent mortality. The text now reads on L25:
    - ➢ From the estimated 7,974 sequoia deaths (95% CI, Bayesian credible interval: 7,555–8,430), corresponding to 30.2% mortality (95% CI: 28.6–31.9%), we found that recent prescribed burns (≤10 years before wildfire) reduced mortality odds by 77% (95% CI: 69–83%).

L31. Just a note that the control treatment / category is not clear from the abstract

- ➢ We thank the reviewer for this comment. We have clarified the reference category in the abstract by explicitly stating that treatment effects are estimated relative to untreated trees within the same grove. Please see L27: "...making treated trees nearly four times more likely to survive relative to untreated trees within the same grove."

L37. Extensive mortality of … associated biodiversity.” Perhaps rephrase.

- ➢ We agree that the original phrasing was imprecise. We have revised the sentence to clarify that high severity fire directly causes tree mortality while threatening associated biodiversity.
  - ➢ The sentence now reads on L37-38:"...forests once considered resistant now experience widespread, high severity fire, causing extensive mortality of iconic overstory trees and threatening associated biodiversity."

L48. Replace ‘defenses’ with ‘adaptations’

- ➢ Great suggestion. Fixed.

L58-60. And also occasionally hostile public opinion!

- ➢ We agree that public opposition to prescribed burning can occur in some contexts. However, the focus of this manuscript is on the ecological and operational challenges faced by managers, which are well documented in the literature and directly relevant to our questions. For this reason, we chose not to expand on public opinion and keep the text as is.

L327. Any bias in the locations of this validation dataset or were these distributed across most of the SEKI?

- ➢ We thank the reviewer for raising this point. The post-fire validation dataset was well distributed spatially, with field observations spanning 14 of the 19 sequoia groves in Sequoia and Kings Canyon national parks. The dataset also included a balanced range of outcomes (753 surviving and 813 dead trees). We have clarified this in the Methods to better describe the spatial coverage and representativeness of the validation data. The new text on L334 now reads: "These field observations were distributed across 14 of 19 SEKI groves and included a balanced range of outcomes (753 surviving and 813 dead trees).”

L342. Unclear what is meant by “user” and “producer”

- ➢ Thanks for noting this ambiguity. In the new revision we clarified the terminology by relating user’s and producer’s accuracy to the more widely used concepts of precision and recall. Please see the new text on L350: “For the Sierra Nevada, 350 the estimated user’s (precision) and producer’s accuracy (recall) ranged from 84.1-86.1%...”

L400. Please state types of uncertainty here or below. i.e. location uncertainty, mortality uncertainty, identity uncertainty?

- ➢ Thanks for requesting this clarification. We now include the types of uncertainty. First, on L400: “The resulting posterior mortality probabilities contain uncertainty from the neural network and its species-specific adjustment to giant sequoias, and also formed the foundation for subsequent analyses, including total mortality estimation and modeling the effects of prescribed burning and forest structure. “
- ➢ Then on L482: "This approach yielded 500 spatially balanced GLMMs that collectively captured uncertainty arising from satellite, sampling, and spatial heterogeneity."

L416. I believe this is a the first mention of the burn treatment indicator variable. Could the authors please add some text earlier that explains how burn treatment is represented in the model approach?

- ➢ That's a good catch. To improve clarity, we now introduce how prescribed burn treatment is represented in the analysis earlier in the Results, within the workflow overview, prior to the detailed model description. Please see the new text:
  - ➢ (Results) L113-115: “Prescribed fire treatment was represented at the tree level as a binary variable distinguishing trees that experienced a prescribed burn prior to wildfire. Step 3 simulated counterfactual prescribed burn histories by modifying the prescribed burn treatment indicator to estimate survival or mortality under alternative management scenarios.”
  - ➢ (Methods) L430-432: “Prescribed burn exposure was represented at the tree level as a binary indicator denoting whether a focal sequoia intersected, or lay within 30 m of, a prescribed burn perimeter occurring within the 10 years preceding wildfire (Supplementary Fig. 5).”

L426. Fig. S9.

- ➢ Fixed. We cleaned up all references to the Supplementary to follow the convention "Supplementary Fig. 2"

L435. Some typo here, repeated 90m

- ➢ Good catch. Please see the new paragraph on lines L435-438 which clarify our definitions and neighborhood sizes of each lidar metric. It now reads: “Canopy density and canopy height heterogeneity were quantified within a 90 m radius surrounding the focal tree, including lidar returns within the focal crown polygon, to capture variation in vegetative material and vertical structure associated with stand age, canopy gaps and openings.”

L513. So this is “prescribed_burn” in the model? Can this be stated?

- ➢ Yes. We have revised the Methods to explicitly clarify that prescribed fire exposure is represented by the binary prescribed_burn indicator in the primary model, defined as whether a focal sequoia intersected a prescribed burn perimeter within the 10 years preceding wildfire.

L107. What are the software packages used for the CNN?

- ➢ The CNN used to generate tree-level mortality probabilities is a previously published model. We have added a description of the software packages used to implement the CNN (Python with TensorFlow and Keras) in the Supplementary Methods and refer readers to Dixon et al. (2023) for full model architecture, training, and inference details.

L148. Please report 95% CI to be consistent with rest of paragraph. Reporting of a single 80% CI for this effect alone is confusing and potentially misleading without additional justification for this choice. Also, in this paragraph, only the prescribe burning effect is translated into a % change in mortality probability, while the others are only presented as log-odds in the figure. Reporting of the raw scale % would be useful for the other effects to compare (e.g. upslope burn).

- ➢ We thank the reviewer for this suggestion. We have revised the Results to report 95% credible intervals consistently for all effects and **removed** the previously reported 80% interval. We present the prescribed burning effect both on the model scale (log-odds) and as an odds reduction for interpretability, while retaining all other covariates on the standardized log-odds scale. Prescribed burning is the primary variable of interest in our causal framework, whereas other covariates are included as adjustment variables; we therefore focus probability-scale interpretation on the treatment effect to avoid over-interpretation of non-causal covariates. The text now clearly reflects this distinction and maintains consistency with Fig. 3.

- ➢ Please see the new text on L149-152: "GLMM ensemble estimates revealed a strong effect of prescribed burning and several weaker or variable effects among structural and environmental covariates (Fig. 3). Recent prescribed burning had a large negative effect on mortality (βprescribed burn = −1.47; 95% CI: [−1.78, −1.17]), corresponding to a 77% reduction in mortality odds (95% CI: [69%, 83%])."

-Also, there is a mix of presentation of credible intervals and confidence intervals here. I might suggest to refer to all as CI, as hardly anyone is interested in the distinction and others find CrI confusing.

- ➢ That's a fair point. We have changed all instances of "CrI" to "CI" throughout the manuscript, since we use Bayesian credible intervals in all cases. To maintain technical accuracy while avoiding confusion, we define CI as "Bayesian credible interval" at its first use in the Abstract (L25) and again as "Bayesian credible interval" in the Results section (L127). All subsequent uses refer simply to "CI" without redefinition.

L269. “The approach is broadly transferable.” I think some more nuanced discussion would be necessary to make this claim. I might suggest that one of the reasons that approach is viable for giant sequoia forests is that canopies are large and forests are relatively sparse, compared to say, a mixed conifer forest where canopies are <3 m wide and tree density is much higher. I

believe the approach may face challenges with resolution in other forests. There were some issues with coordinates not matching tree locations precisely (Quality 2/3) even here. Furthermore, a major strength of the current study is the independent tree-level validation.

- We agree that our original text overgeneralized the transferability of this approach without adequately acknowledging the specific characteristics that made it viable for giant sequoia forests. We have substantially revised the Discussion to provide a more nuanced discussion of transferability. The revised text now explicitly acknowledges that: (1) giant sequoia groves are characterized by large tree crowns (often >10 m diameter) and relatively open canopy structure that are particularly well-suited to 3 m PlanetScope imagery; (2) denser forests with smaller crowns (<3 m) may face resolution constraints requiring finer-scale imagery or alternative approaches; (3) our analysis relied on pre-existing stem location maps integrated with lidar-based crown delineation, a resource that is rarely available in other systems; and (4) where stem maps are unavailable, species identification becomes more challenging. These revisions appropriately contextualize the approach's applicability while maintaining that tree-level methods remain valuable for species-specific studies. Please see the new text on L275-284:

    - “The approach is transferable to other forest systems, with important considerations. Here, we integrated pre-existing stem maps with automated lidar-based crown delineation to enable precise tree-level mortality attribution and neighborhood structure calculation; where stem maps are unavailable, lidar alone can identify individual trees, though species identification becomes more challenging. Giant sequoia groves—characterized by large crowns (often >10 m diameter) and open canopy structure—are well suited to 3 m PlanetScope imagery, whereas denser forests with smaller crowns (<3 m) may require finer-resolution imagery or sub-pixel approaches. Despite these constraints, tree-level analyses enable species-specific inference that provides managers greater confidence in treatment effects. As old-growth trees increasingly face climate-exacerbated disturbance regimes, methods that resolve individual tree outcomes will be essential for guiding conservation strategies.”
